# DEP domain containing 1: a potential oncogenic driver

**DOI:** 10.1186/s40659-025-00643-0

**Published:** 2025-10-08

**Authors:** Yi Cui, Weiwei Ouyang, Daiqin Luo, Youyou Li, Chuan Tian

**Affiliations:** 1 Clinical Research Center, The Affiliated Hospital of Guizhou Medical University, Guiyang, Guizhou China; 2 Department of Oncology, The Affiliated Hospital of Guizhou Medical University, Guiyang, China; 3 Department of Oncology, The Affiliated Cancer Hospital of Guizhou Medical University, Guiyang, Chine; 4 Department of Blood Transfusion, The Affiliated Cancer Hospital of Guizhou Medical University, Guiyang, China

**Keywords:** Pan-cancer, Th2 cells, Functional roles of DEPDC1, Upstream regulators of DEPDC1, Immune cell infiltration, Prognosis

## Abstract

**Background:**

DEP domain-containing protein 1 (DEPDC1) is essential for cell cycle regulation and is increasingly associated with multiple cancers. Accumulating evidence demonstrates that DEPDC1 is significantly upregulated in tumor tissues than in non-malignant tissues across 31 cancer types. DEPDC1 overexpression has been linked to poor prognostic in many cancers, including ACC, KICH, KIRC, KIRP, LGG, LIHC, LUAD, MESO, PAAD, PCPG, SARC, UCEC, and UVM.

**Methods:**

In this study, we analyzed the differential expression of DEPDC1 along with the gene expression profiles associated with it. Specifically, we identified differentially expressed genes (DEGs) associated with higher DEPDC1 expression. These DEGs are involved in key signaling pathways related to cell cycle regulation, DNA damage response, and DNA repair mechanisms across various cancers.

**Results:**

The findings show that heightened DEPDC1 expression correlates with significantly upregulated DEGs associated with critical signaling pathways, including Hedgehog, Hippo, MAPK, MYC, NF-KB, Notch, p27, p53, PI3K/AKT, PLK1, RbE2F, TGF-β, WNT, and MCM across numerous cancers. In addition, elevated DEPDC1 levels positively associate with more TH2 cells, which correlate with poor prognosis. Additionally, a strong relationship was identified between DEPDC1 and genes involved in various immune checkpoints (CD80, CD274, CD276, NRP1, CD160, TNFSF15), chemokines (CXCL8, CXCL5, CXCL10, CCL20, CXCL11, CCL7, CXCL9), chemokine receptor (CCR8, CXCR4, CCR1, CCR3, CXCR6, CCR4, CCR5) genes, and DNA mismatch repair (MMR) processes.

**Conclusions:**

These findings underscore the potential of DEPDC1 as a crucial biomarker in cancer prognosis and the biologic pathways modulation. Future studies are necessary to validate the diagnostic and prognostic implications of DEPDC1, enabling the development of targeted therapeutic interventions targeting this protein, which holds promise for enhancing treatment efficacy in cancer patients.

**Supplementary Information:**

The online version contains supplementary material available at 10.1186/s40659-025-00643-0.

## Introduction

DEPDC1 (DEP domain containing 1) plays a key role in various cancers, suggesting its involvement in tumor development and progression [1]– [2]. It is found in the nucleus and cytoplasm, according to the COMPARTMENTS database (https://compartments.jensenlab.org/search). Among the five transcripts of DEPDC1 identified (refer to Table S1 and Figure S1), two transcripts, ENST00000456315.7 and ENST00000370966.9, are the most frequently observed in normal tissue samples (Figure S2 ). Additionally, DEPDC1 is highly expressed in the testis and EBV-transformed lymphocyte cells, but it has been observed at minimal or non-existent concentrations in various other normal human tissues (Figure S2). Single-cell RNA sequencing (scRNA-seq) data obtained from the Human Protein Atlas (HPA, https://www.proteinatlas.org/) database indicate that DEPDC1 exhibits elevated expression levels in distinct cell types across multiple human tissues (Figure S3).

The oncogenic functions of DEPDC1 have been documented across a variety of tumors, encompassing hepatocellular carcinoma, prostate cancer, renal cell carcinoma, bladder cancer, lung adenocarcinoma, colorectal cancer, gastric cancer, breast cancer, osteosarcoma, oral squamous cell carcinoma and nephroblastoma [3–13]. Although DEPDC1 plays crucial roles in various processes such as the cell cycle [14], maintenance of stemness [15], cell proliferation [3, 8, 13], tumor growth [16], epithelial-mesenchymal transition (EMT) [17], as well as migration, invasion and metastasis [10–13] in specific types of cancer, the molecular mechanisms that govern these functions remain inadequately elucidated. Moreover, the potential of DEPDC1 as a therapeutic target has not been fully explored, warranting extensive investigations into its functional roles across diverse cancer types. Furthermore, the mechanisms governing the upstream regulation of DEPDC1 are not yet fully understood. However, it has been demonstrated that various transcription factors, such as Yin Yang-1 (YY1) and Forkhead box O3 (FOXO3a), alongside specific microRNAs, including miR-26b and miR-20b-3p, play a role in modulating the expression of DEPDC1 [7, 13, 16, 18]. It has been documented that DEPDC1 plays a role in shaping the immune microenvironment associated with natural killer cells [19]. This indicates that further exploration of its possible immunological impacts is warranted. We performed a comprehensive bioinformatics analysis of DEPDC1 in human malignancies, utilizing available fragmented data (see Supplemental text file 1).

This study confirms that DEPDC1 shows high expression in most malignant tumors, and this elevated level negatively affects prognosis, especially in ACC (Adrenocortical carcinoma), KICH (Kidney chromophobe), KIRC (Kidney renal clear cell carcinoma), KIRP (Kidney renal papillary cell carcinoma), LGG (Brain lower grade glioma), LIHC (Liver hepatocellular carcinoma), LUAD (Lung adenocarcinoma), MESO (Mesothelioma), PAAD (Pancreatic adenocarcinoma), PCPG (Pheochromocytoma and Paraganglioma), SARC (Sarcoma), UCEC (Uterine Corpus Endometrial Carcinoma) and UVM (Uveal Melanoma). Evidence suggests that DEPDC1 participates in key pathways such as the cell cycle, DNA damage repair, cell proliferation, immune response and drug sensitivity. The regulatory mechanisms of DEPDC1 include various transcription factors, microRNAs and DNA methylation. The correlation of DEPDC1 with the TH2 (T helper 2 cell)-mediated immunosuppressive microenvironment may facilitate future immunotherapy attempts. DEPDC1 could serve as a biomarker for cancer diagnosis and prognosis and a potential therapeutic target.

## Materials and methods

### Differential expression of DEPDC1

The differential expression of DEPDC1 at mRNA level in cancer is examined using the Cancer Genome Atlas (TCGA) (GDC Data Portal 2.0, analyzed on 01/19/2025, https://portal.gdc.cancer.gov/) and The Genotype-Tissue Expression (GTEx) (GTEx V8 analyzed on 01/19/2025, https://www.gtexportal.org/home/) datasets. The RNA-seq data of cancers are obtained from TCGA datasets, the cancers include ACC, BLCA (Bladder urothelial carcinoma), BRCA (Breast invasive carcinoma), ESAD (Esophageal adenocarcinoma), ESCA (Esophageal carcinoma), KICH, KIRC, KIRP, ESCC (Esophageal squamous-cell carcinomas), MESO, OSCC (Oral squamous cell carcinoma), GBM (Glioblastoma multiforme), HNSC (Head and neck squamous cell carcinoma), DLBC (Lymphoid neoplasm diffuse large B-cell lymphoma), OV (Ovarian serous cystadenocarcinoma), CESC (Cervical squamous cell carcinoma and endocervical adenocarcinoma), CHOL (Cholangio carcinoma), READ (Rectum adenocarcinoma), SARC (Sarcoma), SKCM (Skin cutaneous melanoma), COAD (Colon adenocarcinoma), PCPG (Pheochromocytoma and paraganglioma), LAML (Acute myeloid leukemia), LGG (Brain lower grade glioma), PRAD (Prostate adeno carcinoma), STAD (Stomach adenocarcinoma), LIHC, LUAD, LUSC (Lung squamous cell carcinoma), PAAD, TGCT (Testicular germ cell tumors), THCA (Thyroid carcinoma), THYM (Thymoma), UCEC, UCS (Uterine carcinosarcoma) and UVM. The Toil algorithm is employed to systematically analyze RNA-seq data presented in FPKM (Fragments Per Kilobase Million) format, sourced from the TCGA and GTEx datasets. The mRNA expression levels of DEPDC1 are presented as Log2 (FPKM + 1). To assess the significant differences in DEPDC1 expression between normal/non-tumor tissues and tumor tissues, the Mann Whitney U test was utilized. The levels of statistical significance are denoted as follows: *p < 0.05, **p < 0.01, and ***p < 0.001. Statistical analysis and visualization are performed using Software R (version 4.4.3) and ggplot2 [version 3.4.4] package. DEPDC1 proteomic expression profile in cancer is assessed by data from HPA database. Z-values represent the deviations in standard units from the median across various samples for each specific cancer type.

### Prognostic analysis of DEPDC1

Patients diagnosed with specific types of cancer are categorized according to their expression levels of DEPDC1 (High: 50–100%, Low: 0–50%) for the purpose of conducting survival analyses, which include overall survival, disease-specific survival, and progression-free interval. This stratification is based on data derived from TCGA datasets. Cox regression analysis is used for statistical analysis. For statistical analysis and visualization, R software (version 4.4.3), Survminer [version 0.4.9], and survival [version 3.2–10] packages are employed. Kaplan-Meier (KM) Plotter analysis, including overall survival (OS) and relapse free survival (RFS), is conducted by online Kaplan-Meier analysis tool (https://kmplot.com/analysis/). In addition, time-dependent analysis (1, 3, 5 year) of sensitivity, specificity, and ROC curves are constructed based on DEPDC1 for predicting disease outcome (overall survival, disease specific survival and progression free interval) by using RNA-seq data from TCGA datasets. R software (version 4.4.3), TimeROC (version 0.4) and ggplot2 [version 3.4.4] packages are used for performing statistical analyses and creating visual representations of data.

### DEPDC1-based differential gene expression (DGE) analyses

DESeq2 [version 1.26.0] was employed to evaluate the expression profiles of 59,427 genes in tumor specimens across various cancer types, categorized based on the expression levels of DEPDC1 (High: 50–100%, Low: 0–50%). This study employed RNA sequencing data sourced from The Cancer Genome Atlas (TCGA) databases. The results are represented by Log2 Fold Change (Log2 FC), DEPDC1^High^ vs. DEPDC1^Low^. The genes that significantly distinguish between DEPDC1^High^ and DEPDC1^Low^ groups are chosen based on the condition: |LogFC|>1 and *P* < 0.05. The VennDiagram is then employed to identify the overlapping genes that are upregulated or downregulated across different cancer types.

### Functional roles of DEPDC1

The potential functions of DEPDC1 in various cancers are initially investigated based on scRNA-seq data from CancerSEA dataset (http://biocc.hrbmu.edu.cn/CancerSEA/home.jsp). We then explore the potential roles and underlying mechanisms of DEPDC1 in 33 cancers by performing Gene Set Enrichment Analysis (GSEA)-based pathway analysis, including KEGG, Reactome, Wikipathway (WP) and Biocarta pathways on the above-mentioned DGE data from 33 cancers. The GSEA analyses and visualization are conducted using R (version 4.4.3), clusterProfiler package [version 3.14.3] and ggplot2 [version 3.4.4] package.

### Gene correlation and co-expression analysis

The Spearman correlation coefficient (r) was calculated to assess the relationship between DEPDC1 (Log2 (FPKM + 1)) and the complete set of 59,427 genes (Log2 (FPKM + 1)) derived from TCGA RNA-seq data, for each specific cancer type. This analysis was conducted utilizing the stat package in R version 4.4.3. Genes significantly associated with DEPDC1 in each cancer type are selected based on the condition: |*r* > 0.3| and *P* < 0.05. The VennDiagram is then applied to identify overlapping genes that are either positively (*r* > 0.3 and *P* < 0.05) or negatively (*r*<-0.3 and *P* < 0.05) correlated with DEPDC1 expression across the 36 cancer types. DEPDC1 co-expression genes are identified by generating the overlapping gene set of the DEGs upregulated in High DEPDC1 tumor groups (log2(FC) > 1 and *P* < 0.05), and that are positively correlated with the expression of DEPDC1 (*r* > 0.3 and *P* < 0.05). The co-expression heatmap is then used to show the relative expression of these DEPDC1 co-expression genes stratified by DEPDC1^High^ (50–100%) and DEPDC1^Low^ (0–50%) in the 36 cancer types, respectively. In addition, the pairwise Spearman correlation analysis conducted between DEPDC1 and its co-expressed genes is represented through correlation heatmaps. R (version 4.4.3) and ggplot2 [version 3.4.4] package are used for statistical analysis and visualization.

### Gene set enrichment and survival analysis

The Metascape database (https://metascape.org/gp/index.html#/main/step1) and GeneMANIA database https://genemania.org/) are used to generate of DEPDC1 and its co-expression genes. In addition, functional enrichments, including Reactome pathway, Wikipathway, gene ontology (GO) and KEGG enrichment analysis, are used with the same tool to characterize the potential roles of DEPDC1 and its co-expression genes. Further analysis through Cox regression, we analyze the prognosis of OS, Progression-Free Interval (PFI), Disease-Specific Survival (DSS), Disease-Free Interval (DFI) of DEPDC1 co-expressed genes in pan-carcinoma.

### Upstream regulators of DEPDC1

To find potential transcription factors (TFs) involved in the regulation of DEPDC1, the promoter region of DEPDC1 was analyzed using the e! Ensembl genome browser (http://www.ensembl.org/index.html). The transcription factors (TFs) located upstream of DEPDC1 have been predicted using multiple datasets, one of which is HumanTFD (http://bioinfo.life.hust.edu.cn/HumanTFDB#!/), JASPAR (https://jaspar.genereg.neeg.net/), hTFtarget (http://bioinfo.life.hust.edu.cn/hTFtarget#!/), ENCODE (https://www.encodeproject.org/), CistromeDB Toolkit (http://dbtoolkit.cistrome.org/), Gene card (https://www.genecards.org/), ChIP Atlas (http://chip-atlas.org/), GTRD (https://gtrd.biouml.org/#http://gtrd20-06.biouml.org/) and KnockTF2.0 http://www.licpathway.net/KnockTF/index.php). The TFs predicted by at least 3 datasets are then summarized by VennDiagram. Six miRNA-related databases, including miRWalk (http://mirwalk.umm.uni-heidelberg.de/), miRDB (http://www.mirdb.org/), TargetScanHuman 8.0 (https://www.targetscan.org/vert_80/), DIANA-MicroT-CDS (https://dianalab.ece.uth.gr/html/dianauniverse/index.php?r=microT_CDS), Starbase (https://rnasysu.com/encori/index.php) and DIANA-TarBase v9.0 http://diana.imis.athena-innovation.gr/DianaTools/index.php?r=tarbase/index) are used to predicted the miRNAs with binding sites at the 3’URT of DEPDC1. The miRNAs predicted by at least 3 datasets are selected by VennDiagram. DIANA-Tarbase v8.0 (https://dianalab.e-ce.uth.gr/html/diana/web/index.php?r=tarbasev8/index) and MiRtarbase databases (https://mirtarbase.cuhk.edu.cn/) are experimentally validated microRNA-target interactions databases. The overlapping miRNAs between predicted and experimentally validated miRNA sets are then confirmed by the VennDiagram. The correlation of DEPDC1 expression and DNA methylation is examined based on data from TCGA and Infinium HumanMethylation450 BeadChip array. Statistical analysis and visualization is performed with R (version 4.4.3) and ggplot2 [version 3.4.4] package. The prognostic significance of DEPDC1 methylation is explored using a web tool, MethSurv (https://biit.cs.ut.ee/methsurv/).

### DEPDC1-based immune correlation analysis

This study investigates the association of DEPDC1 with 24 distinct immune cell types across 36 different cancer types, utilizing RNA sequencing data derived from TCGA databases. The immune cell types consist of activated dendritic cells (aDC), immature dendritic cells (iDC), macrophages, B lymphocytes, CD8 T lymphocytes, cytotoxic cells, dendritic cells (DC), eosinophils, mast cells, neutrophils, natural killer (NK) cells categorized as CD56bright and CD56dim, T helper cells, central memory T cells (Tcm), effector memory T cells (Tem), plasmacytoid dendritic cells (pDC), T lymphocytes, follicular helper T cells (Tfh), gamma delta T cells (Tgd), Th1 cells, Th17 cells, Th2 cells and regulatory T cells (Treg). R (version 4.4.3) and GSVA [version 1.34.0] package are used for statistical analysis and visualization. The algorithm for immune infiltration employed within the GSVA package is known as single-sample Gene Set Enrichment Analysis (ssGSEA). The DEPDC1-based immune infiltration is also explored via TIMER2.0 (http://timer.cistrome.org/) online tool. The enrichment score (ES) of immune cells based on DEPDC1 expression is calculated by ssGSEA algorithm. The correlation heatmaps of DEPDC1 and immune cell markers as well as immune checkpoint genes are generated by R (version 4.4.3) and ggplot2 [version 3.4.4] package. The prognostic significance of DEPDC1, in relation to the enrichment of Th2 cells, is examined utilizing the TIMER2.0 online platform alongside KM plotter analysis (https://kmplot.com/analysis/).

### Immunotherapy analysis

A Spearman correlation analysis was conducted to illustrate the relationships between DEPDC1 and the established biomarkers associated with cancer immunotherapy across various cancer types. Tumor mutation burden (TMB) and microsatellite instability (MSI) are widely recognized as important biomarkers for immunotherapy. In the present investigation, we examined the relationships between DEPDC1 and immunotherapy-related biomarkers across various cancer types utilizing data sourced from the TCGA database. Initially, we obtained the gene mutation data for all cancer types available in the TCGA database. Subsequently, we computed the TMB and MSI for each cancer specimen. Then, we conducted an analysis to examine the relationship between the expression levels of DEPDC1 and various factors, including immune checkpoints, chemokines, chemokine receptors, mismatch repair (MMR) genes, as well as genes associated with DNA methylation, TMB and MSI. This analysis was performed utilizing Spearman’s correlation methodology. The outcomes of this study were subsequently illustrated through heatmaps and radar plots for enhanced visualization.

## Results

### High expression and prognostic value of DEPDC1

To evaluate the pan-cancer expression pattern of DEPDC1, we utilized the TCGA and combined TCGA + GTEx datasets to investigate the expression profiles of mRNA and proteins in 33 distinct cancer types. As shown in Figure S4-1A, we observed that DEPDC1 was widely low expressed in human normal tissues, with the top five Higher tissues were the thymus, testis, bone marrow, tonsil and lymph node. Cell lines RNA-seq data showed that the DEPDC1 expression profiles were the low expression of most different cell types using the Human Protein Atlas (HPA) datasets (Figure S4-1B). In addition, DEPDC1 has low expression levels in most tissues from the GTEx databases, except for testis and EBV-transformed lymphocytes cell (Figure S4-1C). The protein of DEPDC1 is mainly expressed in the cerebral cortex, caudate, thyroid gland, esophagus, duodenum, small intestine, rectum, gallbladder, kidney, placenta and skin using the HPA datasets (Figure S4-1D).

**Fig. 1:**
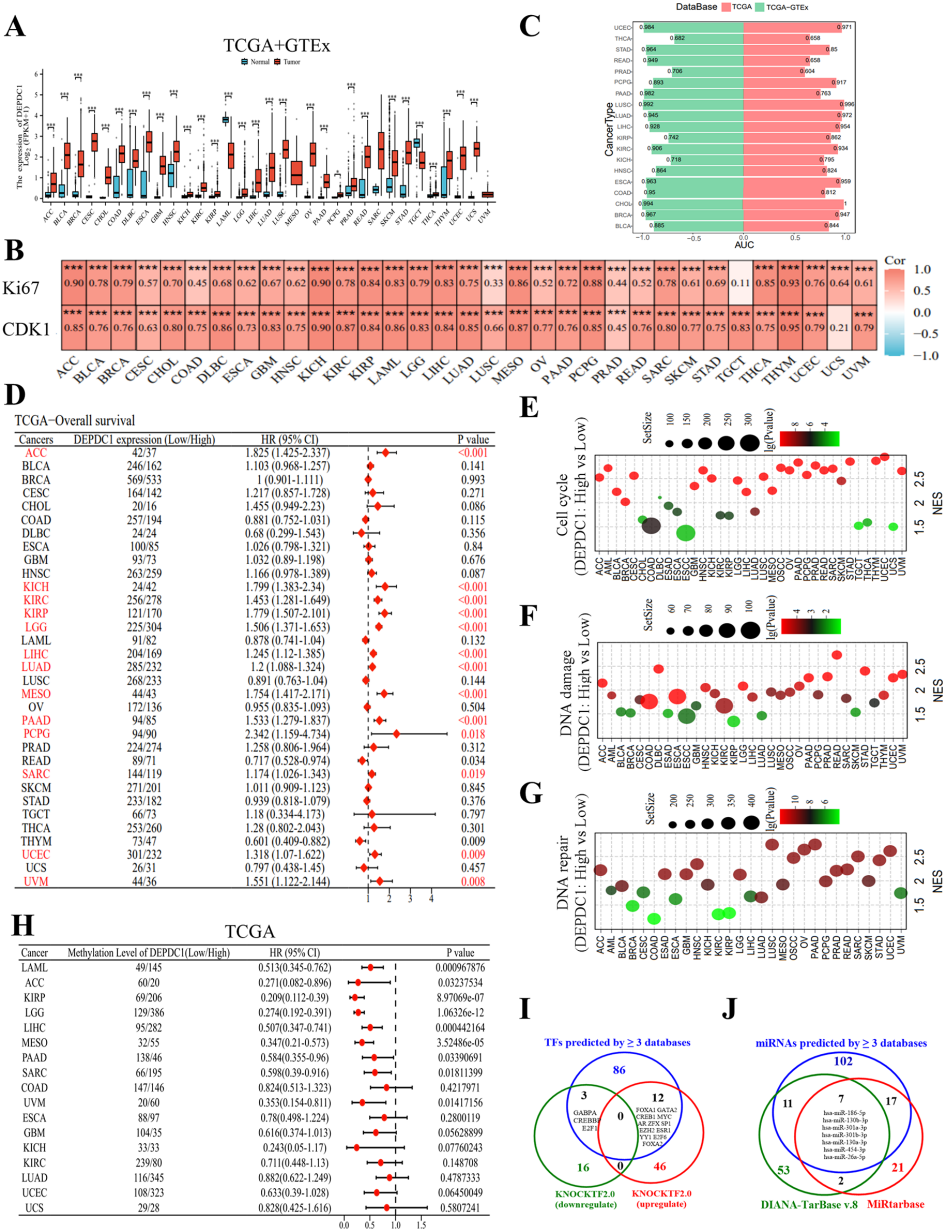
Clinical significance, functional roles and upstream regulators of DEPDC1.(**A**) The Expression profiles of DEPDC1 mRNA in cancers by the analysis of RNA-seq data from TCGA + GTEx database. (**B**) Pan-cancer correlation between DEPDC1 and Ki67, CDK1 using the TCGA database. (**C**) Assess the diagnostic performance of DEPDC1 expression in differentiating between tumor and normal populations through the ROC curve. The horizontal axis illustrates the area beneath the ROC curve (AUC) value, with the blue curve signifying the combined dataset from TCGA-GTEx, while the red curve denotes the TCGA dataset; the vertical axis delineates various tumor types. (**D**) Analysis of overall survival (OS) based on DEPDC1 expression, with patients falling into DEPDC1 high (>median) and DEPDC1 low (< median) groups in each cancer type. GSEAbased analysis of cell cycle-related pathways (**E**), DNA damage (**F**) and DNA repair (**G**) were performed through analyzing DEPDC1-associated differential gene expression data in 36 cancer types. (**H**) Favorable prognostic value of DEPDC1 methylation in cancers determined using the MethSurv database. (**I**) Potential transcription factors responsible for regulating DEPDC1 expression. (**J**) Potential miRNAs targeting DEPDC1. TCGA, The Cancer Genome Atlas; GTEx, Genotype-Tissue Expression. Significance is indicated as follows: *p < 0.05, **p < 0.01, and ***p < 0.001

RNA sequencing data obtained from TCGA and TCGA + GTEx datasets reveal that the mRNA levels of DEPDC1 are markedly elevated in most solid tumors when juxtaposed with normal tissue samples, with the exceptions of TGCT and LAML (Figure S4-2D, Fig. 1A). The analysis of paired samples substantiated the increased expression of DEPDC1 mRNA in tumor tissues relative to normal tissues across 15 distinct cancer types. These include LUSC, BLCA, THCA, BRCA, KIRP, CHOL, COAD, ESCA, HNSC, KICH, KIRC, LUAD, STAD, LIHC and UCEC (Figure S4-2B). Furthermore, DEPDC1 mRNA and protein expression is highly expressed in most tumor cells and tissues (Figure S4-2C and D). In addition, we found that the significant positive correlations of DEPDC1 and Ki-67 (Marker of Proliferation Ki-67), CDK1 (Cyclin Dependent Kinase 1) using the TCGA database in Pan-cancer (Fig. 1B). To further clarify the diagnostic efficacy of DEPDC1, In the TCGA + GTEx and TCGA datasets, our ROC curve analysis revealed that DEPDC1 can effectively distinguish between neoplastic and healthy tissue, suggesting its potential as a universal cancer diagnostic biomarker (Fig. 1C).

The prognostic implications of DEPDC1, indicated by disease-specific survival, overall survival, and progression-free interval, were initially examined in 33 cancer types documented in TCGA. The findings suggest that DEPDC1 may serve as a prognostic biomarker across eight different cancer types, namely ACC, KICH, KIRC, KIRP, LGG, LIHC, LUAD, MESO, PAAD, PCPG, SARC, UCEC and UVM. This is evidenced by the observation that elevated levels of DEPDC1 expression are associated with unfavorable outcomes in terms of DSS, OS and PFI (Figure S5, Fig. 1D, Figure S6). The KM-plotter analysis consistently indicated that elevated levels of DEPDC1 expression correlate with reduced OS, PFI and DSS in various cancer types, including ACC, KIRC, KIRP, LGG, LIHC, LUAD, MESO, PAAD and UVM (Figure S7A-C). Furthermore, time-dependent receiver operating characteristic (ROC) curves validated the prognostic significance of DEPDC1 concerning OS, DSS and PFI in various cancer types including KIRC, ACC, LUAD, KICH, MESO, SARC, KIRP, LGG, PAAD, LIHC, UCEC and UVM (Figure S8A-C). In multiple external datasets, we evaluated the correlation between the expression levels of DEPDC1 and various survival outcomes, including OS, DFS, PFS and RFS utilizing univariate Cox survival analysis. Research shows that low levels of DEPDC1 expression may be associated with better prognoses for patients with ACC, LIHC, LUAD, LGG, KIRC, PAAD and SARC (Figure S9). This indicates that DEPDC1 could be a useful biomarker for predicting patient outcomes, highlighting the need for additional research.

### Functional role of DEPDC1

At the resolution provided by single-cell RNA sequencing (scRNAseq), DEPDC1 exhibited a significant and positive correlation with cellular processes such as the cell cycle, DNA damage response, DNA repair mechanisms and cell proliferation across various cancer types (Figure S10). Utilizing bulk RNA sequencing data derived from TCGA repositories, a differential gene expression (DGE) analysis was conducted on a total of 59,427 genes. This analysis involved comparing samples with high levels of DEPDC1 (greater than the median) against those with low levels of DEPDC1 (less than the median) across 36 distinct cancer types (Additional File 1). In a comprehensive analysis encompassing at least 16 different cancer types, a total of 112 genes exhibited overexpression in tumors characterized by high levels of DEPDC1 compared to those with low DEPDC1 expression. Conversely, six genes demonstrated a significant underexpression in the DEPDC1 ^high^ tumor group when contrasted with the DEPDC1 ^low^ tumor group (Table S2 and S3).

Subsequently, a pathway analysis utilizing GSEA was conducted, encompassing Reactome pathways, KEGG pathways, Biocarta pathways and Wikipathways. This analysis aimed to investigate the biological functions and the relevant signaling pathways influenced by DEPDC1 across the 36 different types of cancer (Additional File 2). According to the scRNAseq data (Figures S10), cell cycle-associated genes were prevalent in DEPDC1^high^ status in all 36 types of cancer (Fig. 1E). Moreover, DEPDC1 is linked to DNA damage in every cancer type except for CHOL, THCA and UCS (Fig. 1F); and the repair of DNA across all cancer types except CHOL, DLBC, ESCC, TGCT, THCA, THYM and UCS (Fig. 1G). Moreover, genes with differential expression linked to signaling pathways such as NOTCH, PI3K/AKT, PLK1, NF-KB, TGF-β, Hedgehog, MYC, MAPK, WNT, HIPPO, P53, P27, MCM and RBE2F exhibited significant enrichment in samples with high levels of DEPDC1 across various cancer types (see Figure S11-1A-I and Figure S11-2A-H). This finding substantiates the observed positive relationship between DEPDC1 and cellular proliferation.

### Gene correlation and co-expression analysis of DEPDC1 in cancer

Utilizing RNA sequencing data (Log2 (FPKM + 1)) obtained from the TCGA database, the Spearman correlation coefficients between DEPDC1 and 59,427 genes across 36 distinct cancer types were investigated (Additional File 3). A comprehensive analysis revealed that a total of 36 genes exhibited a positive correlation with DEPDC1 expression across all 36 types of cancer, with a correlation coefficient (r) exceeding 0.3 and a significance level (P) below 0.05. In stark contrast, no genes were found to have a negative correlation with DEPDC1 expression across any of the cancer types examined (Table S2). In conjunction with the 112 genes that exhibited a correlation with the elevated expression of DEPDC1 across a minimum of 16 cancer types, a total of 23 genes were classified as DEPDC1-co-expressed. This classification was contingent upon the fulfillment of two criteria: a log2 fold change (log2FC) greater than 1 with a significance level of *P* < 0.05, as well as a correlation coefficient (r) exceeding 0.3, also accompanied by a *P* value of less than 0.05 (Table S2). Figure S12 illustrates the correlation heatmaps for DEPDC1 along with 23 co-expression genes across 36 different cancer types. Figure S13-(1–4) illustrates the co-expression heatmaps for the 23 genes across 36 different cancer types, categorized based on DEPDC1 expression levels.

Next, we explored the link between DEPDC1 co-expressed genes and 24 immune cells, finding a significant positive correlation between all tumors and TH2 cells (Figure S14-1-4).

Analysis of single-nucleotide variations (SNVs) revealed that harmful mutations within the 23 co-expression genes exhibited variability across different tumor types. UCEC and SKCM represented the cancer types exhibiting the highest frequency of mutations (Figure S15A). Centromeric Protein E (CENPE) and Kinesin Family Member 4 A (KIF4A) represent the genes with the highest mutation frequency among the 23 co-expressed genes identified in various cancers. Notably, CENPE exhibits an exceptionally elevated mutation rate specifically in mesothelioma. Concomitantly, 23 co-expression genes have almost no mutations in DLBC, CHOL, KICH, MESO, PCPG, THCA, THYM and UVM. The plot displaying single nucleotide variants (SNVs) illustrates the particular conditions surrounding gene mutations (Figure S15B). The predominant type of mutations found in CENPE are missense mutations; however, there is also a significant occurrence of nonsense mutations and splicing mutations.

DNA copy number variations (CNVs) represent a significant aspect of genetic diversity and play a crucial role in the advancement of cancer by influencing gene expression levels. Concurrently, given that CNV exhibits greater stability in comparison to gene expression, it has the potential to serve as a tumor biomarker for the purposes of clinical diagnosis and therapeutic intervention. We examined the possible impact of CNV on the expression of 23 genes that are co-expressed in cancer. CNV analysis results indicated that the primary CNV types in cancers were heterozygous amplification and deletion of 23 co-expressed genes. In addition, the CNV types of TPX2 and RRM2 exhibited instances of homozygous amplification, with a notable increase in the frequency of homozygous amplification observed in uterine carcinosarcoma (UCS) (Figure S16A). In addition, methylation modification serves as a fundamental mechanism for the regulation of gene expression levels. The examination of methylation variations among the 23 co-expressed genes in cancerous tissues compared to normal tissues indicated that these genes exhibited a higher frequency of methylation in BLCA, HNSC, KIRC, LUSC, PAAD, PRAD, and UCEC. Ribonucleotide reductase M2 (RRM2), Targeting protein for Xenopus kinesin-like protein 2 (TPX2) and Topoisomerase II- alpha (TOP2A) were the most frequently methylated genes (Figure S16B). The analysis of the relationship between methylation and mRNA expression across different types of cancer indicated that the expression levels of nearly all genes among the 23 co-expressed genes exhibited a negative correlation with methylation levels. Within the entire set of genes examined, the expression levels of TPX2, RRM2 and Centromere protein I (CENPI) exhibited the most pronounced negative correlation with methylation patterns observed in various cancers (Figure S15C).

Next, we investigated the functional enrichment of genes co-expressed with DEPDC1 in the Metascape database (https://metascape.org/gp/index.html#/main/step1). Gene ontology (GO) and pathway analyses substantiated the participation of DEPDC1 along with its 23 co-expressed genes in processes related to the mitotic cell cycle, the segregation of sister chromatids, the overall cell cycle and the regulation of cell cycle mechanisms (Figure S17A and B). We developed a gene-gene interaction network comprising 23 co-expressed genes to investigate the functional roles of these genes, utilizing the GeneMANIA database for our analysis (https://genemania.org/). The central node, which signifies 23 co-expressed genes, was encircled by 20 additional nodes that denote genes exhibiting significant correlations with the co-expressed genes (Figure S17C). In order to evaluate the prognostic relevance of DEPDC1-associated genes across various cancer types, A univariate Cox proportional hazards regression analysis was conducted using the survival package. The results were subsequently illustrated using a heatmap for enhanced visualization. The findings indicate that every gene analyzed in terms of OS, DSS, PFI and DFI serves as a risk factor across all cancer types examined (Figure S17D-G), with the exception of THYM in the context of OS (Figure S17D).

### Upstream regulators of DEPDC1

DNA methylation serves as a crucial epigenetic mechanism that modulates gene expression. It is noteworthy that the methylation of the CpG site, cg19634693, may contribute to the silencing of DEPDC1. This is evidenced by the inverse relationship observed between the methylation levels of this site and the expression of DEPDC1 mRNA across most cancer types, with the exception of TGCT (Figure S18A-B). Notably, methylation at CpG sites was associated with a positive prognosis in various cancers such as ACC, LAML, KIRP, LGG, LIHC, PAAD, MESO, SARC and UVM. (Fig. 1H).

Eight databases were used to predict the transcription factors (TFs) that might regulate DEPDC1 expression, including CistromeDB Toolkit, JASPAR, HumanTFDB, Genecard, GTRD, hTFtarget, ENCODE and ChIP_Atlas (Additional File 4, Table S4-S5). A total of 101 transcription factors (TFs) were identified through the analysis of three or more databases (Fig. 1I). KnockTF2.0 is a comprehensive database that encompasses RNA sequencing data as well as microarray datasets related to the knockdown or knockout of transcription factors (TFs) achieved through techniques such as siRNA, shRNA or CRISPR in specific tissues or cell types. This database identified a total of 19 transcription factors (TFs) that are responsible for downregulating the expression of DEPDC1, while 58 TFs were found to be associated with its upregulation (Fig. 1I, Figure S19, Additional file 4, Table S4-S5). Within the subset of functionally significant transcription factors (TFs), a total of 15 are of particular interest, as they have been identified by three or more databases (Fig. 1I) to elevate the expression of DEPDC1 mRNA. This group comprises 12 transcription factors: FOXA1, GATA2, CREB1, MYC, AR, ZFX, SP1, EZH2, ESR1, YY1, E2F6 and FOXA2. Additionally, there are three TFs, namely GABPA, CREBBP and E2F1, which are predicted to suppress DEPDC1 mRNA levels.

A total of six databases, namely TargetScanHuman_8.0, miRWalk, DIANA-TarBase v9.0, miRDB, DIANA-MicroT-CDS, and StarBase, were utilized to investigate the microRNAs (miRNAs) that target DEPDC1 (Additional File 5, Tables S6-S7). A total of 137 microRNAs (miRNAs) have significant potential to inhibit DEPDC1, as identified through predictions made by three or more databases (Fig. 1J, Figure S20). Subsequently, an examination of the DIANA-TarBase v8.0 and MiRtarbase databases was performed to pinpoint 111 miRNAs that have been experimentally validated as targeting DEPDC1(Additional File 5, Figure S20). Seven miRNAs were found to overlap in the correlative and functional analyses (Fig. 1J).

### Immune infiltration related to DEPDC1 in cancer

**Fig. 2:**
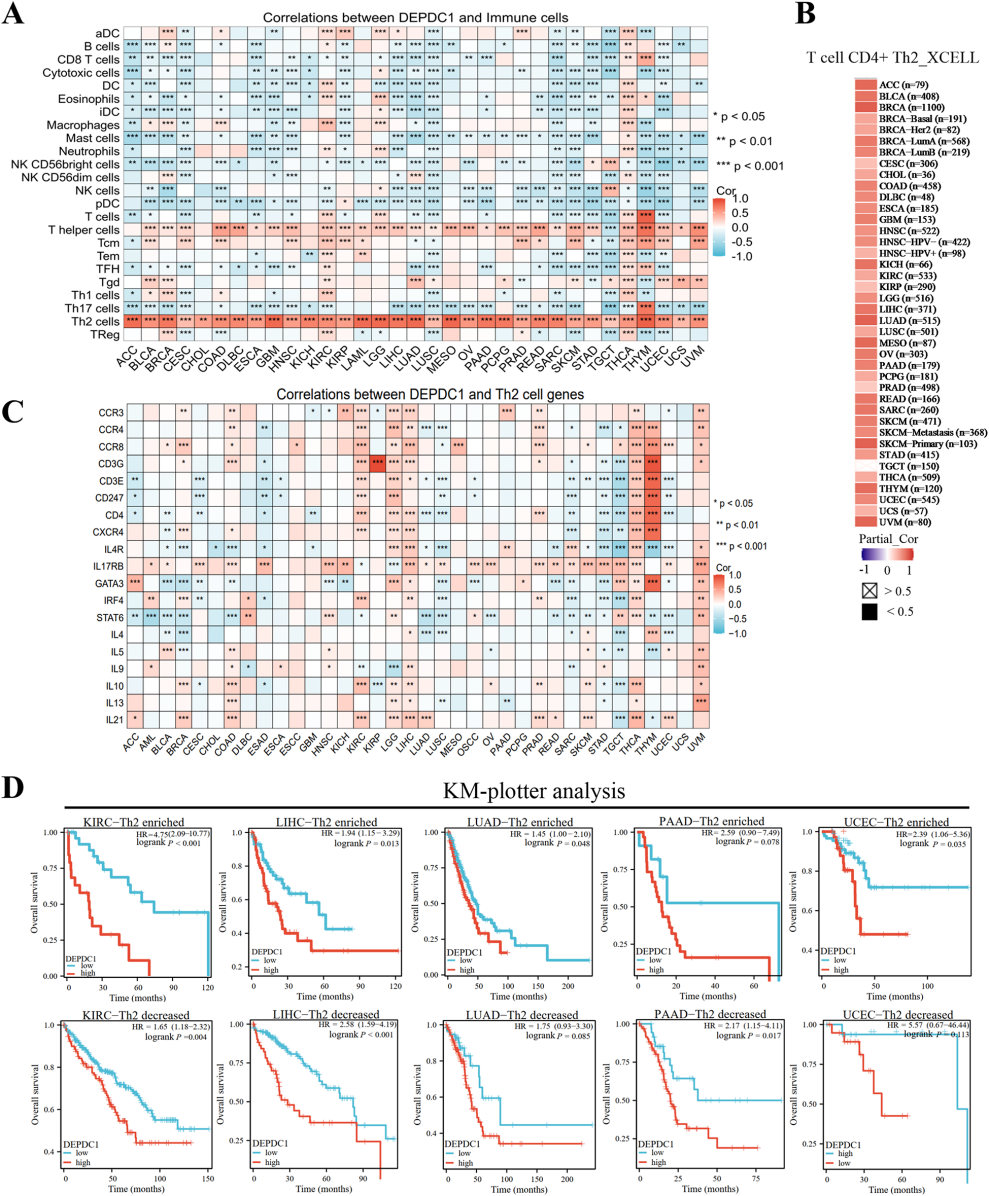
(**A**) Correlation of DEPDC1 with 24 immune cell types in 36 cancers. (**B**) Extraction of XCELL datasets indicate a significantly positive correlation of DEPDC1 expression and Th2 cell enrichment in these 36 cancer types. (**C**) Correlation of DEPDC1 and Th2 cell markers genes presented as heatmaps. Statistical significance is shown as *p < 0.05, **p < 0.01, and ***p < 0.001. (**D**) Survival impact of DEPDC1 expression in cancers with enhanced or reduced Th2 cell level determined by means of the KM-plotter online tool

To comprehend how DEPDC1 regulates the immune system in cancers, researchers investigated its correlation with the infiltration of 24 immune cell types in the TCGA. In all 36 cancer types, DEPDC1 showed a positive correlation with the enrichment of CD4 + T lymphocytes of the TH2 (*r* > 0.3, *P* < 0.05). Alternatively, the infiltration of other immune cells showed either a positive or negative correlation with DEPDC1, depending on the cancer type (Fig. 2A, Figure S21). Similarly, TIMER2.0 results indicated a positive link between DEPDC1 and TH2 enrichment in cancers (Fig. 2B). Moreover, the enrichment score (ES) for TH2 cells is higher in DEPDC1^high^ samples than in DEPDC1^low^ samples across 36 cancer types (Figure S22). In certain cancers, DEPDC1 showed a significant association with many TH2 cell markers (CXCR4, CCR3, CD4, IL4R, IL13, CCR4, CCR8, CATA3, IRF4, CD3G, IL4, IL5, CD3E, CD247, IL17RB, STAT6, IL9, IL10 and IL21), including KIRC, LGG, LIHC, PRAD, THCA and THYM (Fig. 2C).

Furthermore, we evaluated the prognostic value of TH2 cells based on DEPDC1 expression. The enrichment of TH2 cells was linked to unfavorable outcomes in cancers like KIRC, HNSC, KIRP, KICH, LIHC, VUM, THYM and PCPG, depending on DEPDC1 expression (Figure S23). Moreover, elevated DEPDC1 mRNA was a poor outcome predictor for patients with high intratumoral TH2 cell levels in PAAD, LICH, SARC, LUAD and UCEC. The predictive significance of DEPDC1 was absent in LUAD and UCEC tumors with low TH2 levels, indicating a notable interaction between DEPDC1 and TH2 cell infiltration (Fig. 2D).

### Immunomodulatory factors and drug sensitivity analysis

**Fig. 3:**
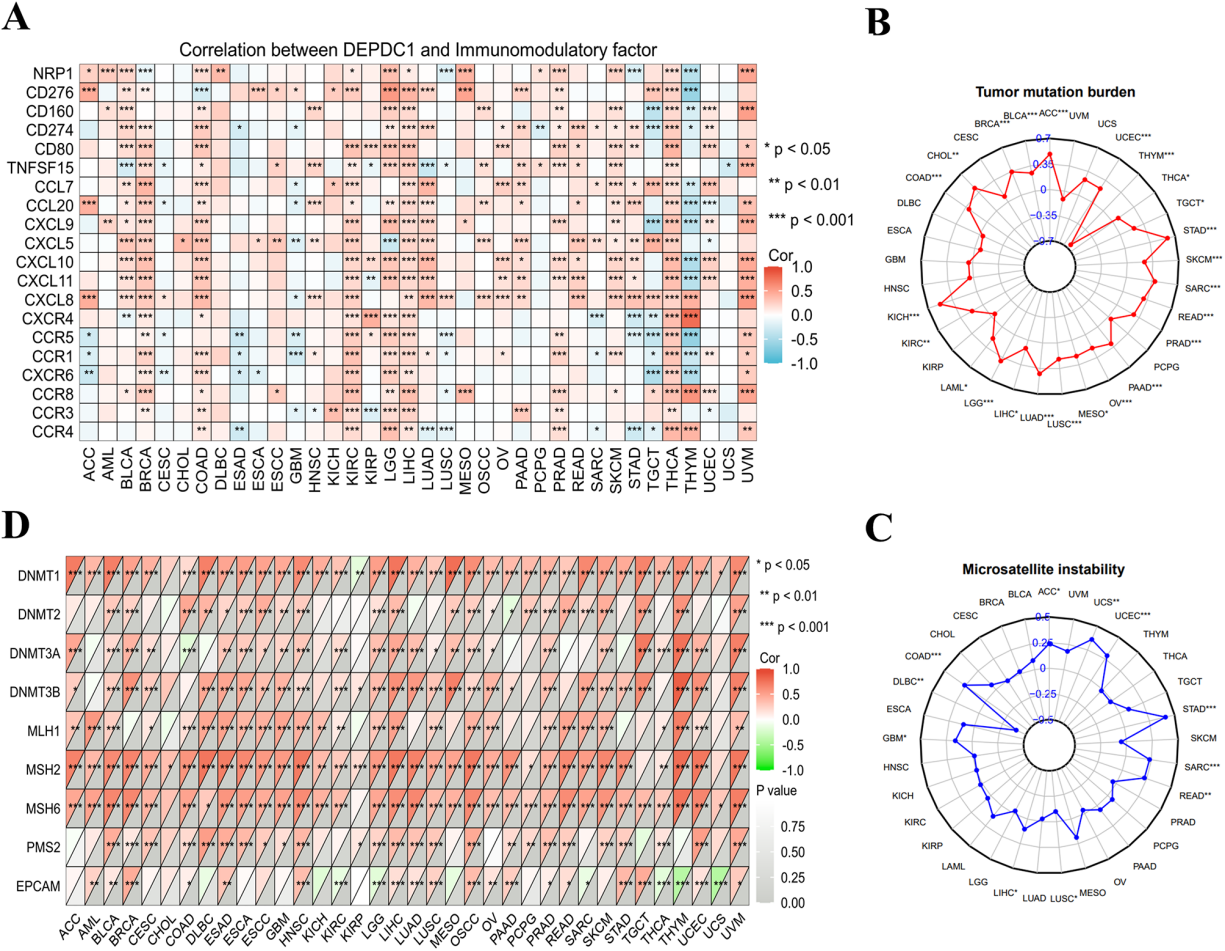
(**A**) The correlation between DEPDC1 expression and immune checkpoint, chemokines, chemokine receptors gene expression in cancers. (**B**) The radar chart displayed the correlation between DEPDC1 expression and TMB (Tumor Mutation Burden). (**C**) The radar chart displayed the correlation between DEPDC1 expression and MSI (Microsatellite Instability). (**D**) Relationship between the expression of Four methyltransferase genes, Five MMR genes and DEPDC1 in the pan-cancer

Displayed were the connections between DEPDC1 and immunomodulatory factors in different types of cancer (Fig. 3A, Figure S24-1 and 2). Moreover, DEPDC1 showed positive correlations with certain immune checkpoint (NRP1, CD276, CD160, CD274, CD80 and TNFSF15), chemokines (CXCL5, CCL7, CXCL10, CCL20, CXCL9, CXCL11 and CXCL8) and chemokine receptors (CXCR4, CCR5, CCR1, CXCR6, CCR8, CCR3 and CCR4) genes in various cancers, particularly with immune checkpoints (LGG, LIHC, MESO, PRAD, THCA and UVM), chemokines (COAD, BLCA, LIHC, BRCA, LUAD, KIRC, SKCM, READ and THCA), and chemokine receptors (KIRC, LGG, LIHC and THCA) (Fig. 3A). The relationship between DEPDC1 expression and TMB (Tumor Mutation Burden) and MIS (Microsatellite Instability) was further evaluated to comprehend DEPDC1’s role in forecasting the effectiveness of immune checkpoint inhibitors (ICIs). ACC, UCEC, THCA, TGCT, STAD, SKCM, SARC, READ, PRAD, PAAD, OV, MESO, LUSC, LUAD, LIHC, LGG, LAML, KIRC, KICH, COAD, CHOL, BRCA and BLCA showed positive correlations with TMB, while THYM exhibited negative correlations (Fig. 3B). Furthermore, a positive correlation between DEPDC1 expression and MSI was found in ACC, UCS, UCEC, STAD, SARC, READ, LIHC, GBM and COAD, while a negative correlation was observed in LUSC and DLBC (Fig. 3C). Our findings indicated that DEPDC1 could potentially forecast the effectiveness of ICIs in the relevant cancers.

The relationship between DEPDC1 expression levels and mutations in five MMR genes, such as MLH1, PMS2, MSH2, EPCAM and MSH6 was examined to evaluate DEPDC1’s role in tumorigenesis. According to the findings, the MMR genes were correlated with DEPDC1 and this correlation was found in the majority of cancer types (Fig. 3D). Especially in the UVM, UCEC, SKCM, READ, PRAD, OSCC, LUSC, LIHC, LGG, HNSC, GBM, ESCC, ESCA, ESAD, DLBC, COAD, CESC and BLCA, DEPDC1 expression showed a significant positive correlation with the genes MLH1, MSH2, MSH6 and PMS2. In UCS, THYM, THCA, SARC, LGG, and KIRC, a negative correlation with the EPCAM gene was observed. Moreover, irregular DNA methylation is a significant factor in promoting tumor development and is considered a promising biomarker for diagnosis, treatment, and prognosis. Additional research was carried out to explore the relationship between the expression of four methyltransferase genes (DNMT1, DNMT2, DNMT3A, DNMT3B) and DEPDC1 in different types of cancer (Fig. 3D). The findings indicated that DEPDC1 expression was closely linked with at least one methyltransferase gene in 31 of the 36 cancer types (not in COAD, AML, CHOL, KICH and UCS).

Furthermore, we examined the connection between DEPDC1 and drug sensitivity using the GDSC1 or 2 (Genomics of Drug Sensitivity in Cancer 1 or 2, http://www.cancerrxgene.org/), PRISM (https://depmap.org/repurposing) and CTRP (Cancer Therapeutic Response Portal, https://portals.broadinstitute.org/ctrp.v2.1/) databases to investigate DEPDC1’s role in other cancer treatments. The results of PRISM and CTRP databases show that DEPDC1 is significantly negatively correlated with the Clofarabine, Etoposide and LY2183240, while GDSC1 and GDSC2 are significantly positively correlated with PD0325901, Selumetinib, Trametinib and VX11e in pan-cancer (Figure S25A). The Cmap database (Connectivity map, https://clue.io/) is employed in pan-cancer research to contrast DEPDC1-associated characteristics with cMAP gene features. Similarity scores of 1288 compounds were obtained. Compounds with lower fractions may inhibit DEPDC1-mediated cancer-promoting effects. As shown in Figure S25B, in some tumors, arachidonyltrifluoromethance and MS.275 may reverse molecular signatures caused by dysregulation of DEPDC1 expression, thus counteracting its mediated cancer-promoting effects.

## Discussion

DEPDC1 (DEP domain containing 1) has been identified as a negative prognostic indicator in various solid tumors, such as breast cancer, renal cell carcinoma, HCC, PCa, bladder cancer, LUAD and gastric cancer ^(3–6,8,9,13)^. Recent studies have confirmed that DEPDC1 is overexpressed in gastric cancer, breast cancer, osteosarcoma, and hepatocellular carcinoma at both mRNA and protein levels, and its elevation is linked to unfavorable clinical outcomes in patients with these cancers ^(8–10,13)^. However, the clinical significance of DEPDC1 in other cancer types has remained largely unknown. Our in silico differential expression analysis indicated that DEPDC1 is highly expressed in the majority of solid tumors. Additionally, survival analyses pointed out the substantial potential of DEPDC1 as a detrimental biomarker in ACC, BLCA, COAD, HNSC, KICH, LGG, LIHC, LUAD, KIRC, KIRP, MESO, PAAD, PCPG, SARC, THCA, UCEC, UVM. As far as we know, there have been no reports on the clinical correlation between DEPDC1 and the prognosis of ACC, BLCA, COAD, HNSC, KICH, KIRP, LGG, MESO, PCPG, SARC, UCEC and UVM.

DEPDC1 acts as a tumor promoter in various cancers ^[4–6,8−17]^. HCC is where the functional roles of DEPDC1 have been most effectively shown among all reported cancer types. DEPDC1 serves as a predictor for the prognosis of patients with hepatocellular carcinoma and plays a role in regulating tumor growth and metastasis, DEPDC1 expression is positively correlated with WNT/β-catenin signal pathway [20]. Accordingly, we found that DEPDC1 promoted HCC migration and invasion via Wnt/β-catenin signaling pathway and EMT [21]. Furthermore, the DEPDC1/E2F signaling pathway has been associated with prostate cancer growth. DEPDC1 has been found to play similar roles in various other cancers, including renal cell carcinoma, bladder cancer, lung adenocarcinoma, gastric cancer, breast cancer and osteosarcoma ^[4–6,8−10]^. Our analysis across various cancers shows that DEPDC1 is generally connected to the cell cycle, proliferation, DNA damage, and repair, as indicated in Figure S10, supporting previous research [1, 8, 14]. Cell cycle-related genes were found to be upregulated in DEPDC1^high^ tumors across all 36 cancer types. Moreover, DEPDC1 had a positive correlation with DNA damage and repair in the majority of tumors. DEPDC1 was also found to have strong positive correlations with pathways related to tropic signals in most cancer types.

Currently, there is proof that the transcription factor FOXO3a-regulated DEPDC1 enhances the malignant progression of nephroblastoma through the Wnt/β-catenin signaling pathway [18]. Furthermore, DEPDC1, negatively regulated by miR-26b, facilitates cell proliferation via the up-regulation of FOXM1 expression in TNBC [16]. DEPDC1 is also targeted by miR-130b-3p in LUAD and miR-7-5p in HCC, both of which show antiproliferative effects [13, 22]. In this study, we discovered more transcription factors and miRNAs that influence DEPDC1 mRNA expression. Additionally, methylation at the CpG sites cg19634693 was found to be negatively associated with DEPDC1 expression and had beneficial prognostic implications for multiple cancers (Fig. 1H, Figure S18).

Tumor formation and progression are complex and dynamic, relying on the communication between the tumor and its surrounding immune environment. Interestingly, DEPDC1 was associated with an increase in TH2 cells across all 36 types of cancer. TH2 cells are strongly linked to chronic inflammation, which is thought to promote tumor growth [23]. Notably, additional survival analyses suggest that the negative prognostic impact of DEPDC1 relies on TH2 cells in KIRC, LIHC, LUAD, and PAAD. DEPDC1 is also significantly associated with a majority of immune checkpoint genes (CD80, CD274, CD276, NRP1, CD160, TNFSF15) in LGG, LIHC, MESO, PRAD, THCA and UVM, and with chemokine genes (CXCL8, CXCL5, CXCL10, CCL20, CXCL11, CCL7, CXCL9) in LIHC, BLCA, LUAD, BRCA, READ, COAD, SKCM, KIRC and THCA, along with chemokine receptor genes (CCR8, CXCR4, CCR1, CCR3, CXCR6, CCR4, CCR5) in LGG, KIRC, LIHC, LGG and THCA, indicating that DEPDC1 may be involved in fostering an immunosuppressive microenvironment. Research involving immunohistochemical and proteomic analysis of DEPDC1 protein levels in different cancers is required to confirm the theory that DEPDC1 expression influences tumor progression both prognostically and immunologically.

Currently, there are no drugs that specifically target DEPDC1. Notably, dual EZH2/CDK4/6 blockade inhibited tumor growth and DEPDC1 towards a more differentiated state [24]. Interestingly, 67 chemicals reduce the expression of DEPDC1 at the mRNA or protein levels, or they decrease the phosphorylation of the DEPDC1 protein in humans, according to the Comparative Toxicogenomics Database (CTD, http://ctdbase.org/) (Additional File 6). Nonetheless, these drugs have not yet been assessed for their possible anticancer properties. It would be especially fascinating to evaluate these hypothetical DEPDC1 inhibitors for their effects on the TH2-driven immunosuppressive microenvironment, both independently and in conjunction with current immune checkpoint, chemokine, and chemokine receptor inhibitors.

## Conclusion

DEPDC1 is significantly overexpressed in most cancers, and this excessive expression negatively affects prognosis, particularly in ACC, KICH, KIRC, KIRP, LGG, LIHC, LUAD, MESO, PAAD, PCPG, SARC, UCEC and UVM. Evidence indicates that DEPDC1 plays a role in crucial signaling pathways related to the cell cycle, DNA damage and repair, cell proliferation, immune response, and drug sensitivity. The regulation of DEPDC1 expression is influenced by different transcription factors, miRNAs and DNA methylation. The association of DEPDC1 with a TH2-dominant immunosuppressive tumor microenvironment might inspire future efforts to target DEPDC1 in immunotherapy contexts. It may serve not only as a promising biomarker for cancer diagnosis and prognosis but also as a potential therapeutic target, paving the way for personalized treatment strategies for cancer patients.

## Electronic Supplementary Material

Below is the link to the electronic supplementary material.

Supplementary Material 1

Supplementary Material 2

Supplementary Material 3

Supplementary Material 4

Supplementary Material 5

Supplementary Material 6

Supplementary Material 7

Supplementary Material 8

## Data Availability

The datasets used or analyzed during the current study are available from the corresponding author on reasonable request.

## Abbreviations

DEPDC1: DEP domain-containing protein 1
PCa: Prostate cancer
ACC: Adrenocortical carcinoma
BLCA: Bladder urothelial carcinoma
BRCA: Breast invasive carcinoma
CESC: Cervical squamous cell carcinoma and endocervical adenocarcinoma
CHOL: Cholangio carcinoma
COAD: Colon adenocarcinoma
DLBC: Lymphoid neoplasm diffuse large B-cell lymphoma
ESAD: Esophageal adenocarcinoma
ESCA: Esophageal carcinoma
ESCC: Esophageal squamous-cell carcinomas
GBM: Glioblastoma multiforme
HNSC: Head and neck squamous cell carcinoma
KICH: Kidney Chromophobe
KIRC: Kidney renal clear cell carcinoma
KIRP: Kidney renal papillary cell carcinoma
LAML: Acute myeloid leukemia
LGG: Brain lower grade glioma
LIHC: Liver hepatocellular carcinoma
LUAD: Lung adenocarcinoma
LUSC: Lung squamous cell carcinoma
MESO: Mesothelioma
OSCC: Oral squamous cell carcinoma
OV: Ovarian serous cystadenocarcinoma
PAAD: Pancreatic adenocarcinoma
PCPG: Pheochromocytoma and Paraganglioma
PRAD: Prostate adenocarcinoma
READ: Rectum adenocarcinoma
SARC: Sarcoma
SKCM: Skin cutaneous melanoma
STAD: Stomach adenocarcinoma
TGCT: Testicular germ cell tumors
THCA: Thyroid carcinoma
THYM: Thymoma
UCEC: Uterine corpus endometrial carcinoma
UCS: Uterine carcinosarcoma
UVM: Uveal melanoma
DSS: Disease specific survival
OS: Overall Survival
PFI: Progression Free Interval
RFS: Relapse free survival
EMT: Eepithelial-mesenchymal transition
GSEA: Gene set enrichment analysis
NES: Normalized enrichment score
WP: Wikipathway
TFs: Transcription factors
miRNAs: MicroRNAs
