# Supplementary material for: DEP domain containing 1: a potential oncogenic driver
Source: Biol Res. 2025 Oct 8;58:64. doi: 10.1186/s40659-025-00643-0 (PMC12505635; doi:10.1186/s40659-025-00643-0)
Supplement: Supplementary file 8 — Supplementary Material 8 [file 40659_2025_643_MOESM8_ESM.docx]

**Table S1. Transcripts of DEPDC1 in Human**

| Transcript ID | Biotype | Name | bp | Translation ID | Protein |
| --- | --- | --- | --- | --- | --- |
| ENST00000456315.7 | Protein coding | DEPDC1-202 | 5299 | ENSP00000412292.2 | 811aa |
| ENST00000370966.9 | Protein coding | DEPDC1-201 | 4586 | ENSP00000360005.5 | 527aa |
| ENST00000525124.1 | Protein coding | DEPDC1-205 | 582 | ENSP00000431477.1 | 34aa |
| ENST00000489862.1 | Nonsense mediated decay | DEPDC1-204 | 1613 | ENSP00000436464.1 | 308aa |
| ENST00000488146.5 | Retained intron | DEPDC1-203 | 2658 | - | No protein |

Note: the information here is retrieved from e!Ensembl database (<https://www>.ensembl.org/index.

html).

**Table S2. Differentially expressed genes (DEGs) based on DEPDC1 expression and the co-expression genes of DEPDC1 in cancers**

| **Cancers** | **Total no. of genes** | **DEGs (DEPDC1: High vs**  **Low, \|log2(FC)\|>1 & P <0.05)** | | | **Genes significantly associated with DEPDC1 (Spearman \|r>0.3\| & P<0.05)** | | | **Co-expression genes of**  **DEPDC1(r>0.3, P<0.05**  **& log2 FC>1, P<0.05)** |
| --- | --- | --- | --- | --- | --- | --- | --- | --- |
|  |  | **Total** | High/Low | Overlap genes | **Total** | Positive/  Negative | Overlap genes |  |
|  |  |  |  | High/Low |  |  | Positive/  Negative |  |
| ACC | **59427:**  Protein coding,  (19934),  lncRNA (16876),  miRNA  (1879),  Others  (20738) | **3422** | 2076/1346 | **High: 112**  1 (35 cancers)  1 (27 cancers)  6 (25 cancers)  1 (24 cancers)  9 (23 cancers)  6 (22 cancers)  6 (21 cancers)  12 (20 cancers)  15 (19 cancers)  19 (18 cancers)  17 (17 cancers)  19 (16 cancers)  **Low: 6**  1 (17 cancers)  5 (16 cancers) | **6278** | 5501/777 | **Positive: 36**  DEPDC1  PRR11  HMMR  ORC1  RAD54L  AURKA  TPX2  KIF4A  CDKN3  GINS1  CENPI  FOXM1  FBXO5  KIF20A  LMNB1  NEK2  TMPO  TOP2A  CDCA8  CKAP2  KIF23  KIF20B  CENPE  KIF2C  NUF2  CCNA2  NCAPG2  SPC25  SGO2  KIF15  MAD2L1  E2F7  RRM2  HASPIN  PARPBP  FAM72A  **Negative: 0** | **Genes: 23**  DEPDC1  HMMR  TOP2A  CCNA2  NEK2  NUF2  KIF4A  KIF23  CENPE  KIF20A  KIF15  FOXM1  RRM2  TPX2  CDCA8  CDKN3  CENPI  HASPIN  E2F7  FAM72A  KIF2C  MAD2L1  ORC1 |
| AML/  LAML |  | **1913** | 1524/389 |  | **2877** | 2305/572 |  |  |
| BLCA |  | **3877** | 2517/1360 |  | **3560** | 2689/871 |  |  |
| BRCA |  | **3767** | 3078/689 |  | **3868** | 1705/2163 |  |  |
| CESC |  | **1216** | 332/884 |  | **1712** | 1659/53 |  |  |
| CHOL |  | **1802** | 1000/802 |  | **2741** | 1823/918 |  |  |
| COAD |  | **5721** | 5373/348 |  | **3653** | 2749/904 |  |  |
| DLBC |  | **3592** | 807/2785 |  | **10168** | 6977/3191 |  |  |
| ESAD |  | **3717** | 1965/1752 |  | **4643** | 3365/1278 |  |  |
| ESCA |  | **3002** | 2292/710 |  | **2106** | 2038/68 |  |  |
| ESCC |  | **2212** | 1777/435 |  | **3522** | 3258/264 |  |  |
| GBM |  | **975** | 735/240 |  | **2450** | 2095/355 |  |  |
| HNSC |  | **1165** | 561/604 |  | **3523** | 3408/115 |  |  |
| KICH |  | **1898** | 1127/771 |  | **3723** | 3177/546 |  |  |
| KIRC |  | **2152** | 1605/547 |  | **1031** | 905/126 |  |  |
| KIRP |  | **2164** | 1926/238 |  | **1247** | 1059/188 |  |  |
| LGG |  | **1534** | 1299/235 |  | **3570** | 2782/788 |  |  |
| LIHC |  | **3908** | 3273/635 |  | **7872** | 7656/216 |  |  |
| LUAD |  | **5905** | 4555/1350 |  | **4555** | 2513/2042 |  |  |
| LUSC |  | **979** | 447/532 |  | **1365** | 1118/247 |  |  |
| MESO |  | **3119** | 1477/1642 |  | **4656** | 3063/1593 |  |  |
| OSCC |  | **1212** | 629/583 |  | **3291** | 3196/95 |  |  |
| OV |  | **686** | 458/228 |  | **1134** | 1084/50 |  |  |
| PAAD |  | **1981** | 692/1289 |  | **3005** | 2002/1003 |  |  |
| PCPG |  | **1226** | 505/721 |  | **815** | 677/138 |  |  |
| PRAD |  | **720** | 365/355 |  | **1448** | 1391/57 |  |  |
| READ |  | **1020** | 282/938 |  | **3523** | 2950/573 |  |  |
| SARC |  | **4095** | 1905/2190 |  | **2827** | 2270/557 |  |  |
| SKCM |  | **1838** | 690/1148 |  | **4811** | 4646/165 |  |  |
| STAD |  | **2784** | 711/2073 |  | **4657** | 2744/1913 |  |  |
| TGCT |  | **7692** | 3680/4012 |  | **11627** | 6211/5416 |  |  |
| THCA |  | **1441** | 1328/113 |  | **1452** | 1396/56 |  |  |
| THYM |  | **8178** | 1688/6490 |  | **12884** | 6888/5996 |  |  |
| UCEC |  | **3490** | 1689/1801 |  | **5203** | 4388/815 |  |  |
| UCS |  | **2734** | **1079/**1655 |  | **3195** | 1328/1867 |  |  |
| UVM |  | **2497** | 1261/1236 |  | **14597** | 13212/1385 |  |  |

**Table S3. Overlap genes of Differentially expressed genes in cancers**

| **High expression genes** | **Total no. of genes or cancers** | **Overlap genes** | **Cancers types** |
| --- | --- | --- | --- |
|  | 1 (35 cancers) | DEPDC1 | ACC. AML. BLCA. BRCA. CESC. CHOL. COAD. DLBC. ESAD. ESCA. ESCC. GBM. HNSC. KICH. KIRC. KIRP. LGG. LIHC. LUAD. LUSC. MESO. OSCC. OV. PAAD. PCPG. PRAD. READ. SARC. SKCM. STAD. TGCT. THCA. THYM. UCEC. UVM. |
|  | 1 (27 cancers) | HMMR | ACC. AML. BLCA. BRCA. CHOL. COAD. ESAD. ESCA. ESCC. GBM. KICH. KIRC. KIRP. LGG. LIHC. LUAD. MESO. PAAD. PCPG. PRAD. SARC. SKCM. TGCT. THCA. THYM. UCEC. UVM. |
|  | 1 (25 cancers) | DEPDC1-AS1 | ACC. AML. BLCA. BRCA. CHOL. COAD. ESAD. GBM. KICH. KIRC. KIRP. LGG. LIHC. LUAD. LUSC. MESO. OSCC. OV. PAAD. PRAD. READ. SARC. SKCM. THYM. UCEC. |
|  | 1 (25 cancers) | KIF14 | ACC. AML. BLCA. BRCA. CHOL. DLBC. GBM. KICH. KIRC. KIRP. LGG. LIHC. LUAD. MESO. OSCC. PAAD. PCPG. PRAD. SARC. SKCM. STAD. THCA. THYM. UCEC. UVM. |
|  | 1 (25 cancers) | NCAPG | ACC. AML. BLCA. BRCA. CHOL. DLBC. GBM. KICH. KIRC. KIRP. LGG. LIHC. LUAD. MESO. OV. PAAD. PCPG. PRAD. SARC. SKCM. STAD. THCA. THYM. UCEC. UVM. |
|  | 1 (25 cancers) | TOP2A | ACC. AML. BLCA. BRCA. CHOL. GBM. KICH. KIRC. KIRP. LGG. LIHC. LUAD. MESO. OV. PAAD. PCPG. PRAD. SARC. SKCM. STAD. THCA. THYM. UCEC. UCS. UVM. |
|  | 1 (25 cancers) | PBK | ACC. AML. BRCA. CHOL. COAD. GBM. KICH. KIRC. KIRP. LGG. LIHC. LUAD. MESO. OV. PAAD. PCPG. PRAD. READ. SARC. SKCM. STAD. THCA. THYM. UCEC. UVM. |
|  | 1 (25 cancers) | AL138789.1 | AML. BLCA. BRCA. CESC. CHOL. COAD. DLBC. ESAD. ESCA. ESCC. KIRC. KIRP. LGG. LIHC. LUAD. OV. PAAD. PRAD. READ. SARC. SKCM. STAD. THYM. UCEC. UCS. |
|  | 1 (24 cancers) | CCNA2 | ACC. AML. BLCA. BRCA. CHOL. GBM. KICH. KIRC. KIRP. LGG. LIHC. LUAD. MESO. OV. PAAD. PCPG. PRAD. SARC. SKCM. TGCT. THCA. THYM. UCEC. UVM. |
|  | 1 (23 cancers) | BUB1B | ACC. AML. BLCA. BRCA. CHOL. DLBC. GBM. KICH. KIRC. KIRP. LGG. LIHC. LUAD. MESO. OSCC. PAAD. PCPG. PRAD. SARC. STAD. THYM. UCEC. UVM. |
|  | 1 (23 cancers) | ANLN | ACC. AML. BLCA. BRCA. CHOL. DLBC. KICH. KIRC. KIRP. LIHC. LUAD. MESO. OSCC. PAAD. PCPG. PRAD. SARC. SKCM. STAD. THCA. THYM. UCEC. UVM. |
|  | 1 (23 cancers) | NEK2 | ACC. AML. BLCA. BRCA. CHOL. GBM. KICH. KIRC. KIRP. LGG. LIHC. LUAD. MESO. OSCC. PAAD. PCPG. PRAD. SARC. SKCM. THCA. THYM. UCEC. UVM. |
|  | 1 (23 cancers) | DLGAP5 | ACC. AML. BLCA. BRCA. CHOL. GBM. KICH. KIRC. KIRP. LGG. LIHC. LUAD. MESO. OV. PAAD. PCPG. PRAD. SARC. SKCM. THCA. THYM. UCEC. UVM. |
|  | 2 (23 cancers) | TTK CENPF | ACC. AML. BLCA. BRCA. CHOL. GBM. KICH. KIRC. KIRP. LGG. LIHC. LUAD. MESO. PAAD. PCPG. PRAD. SARC. SKCM. STAD. THCA. THYM. UCEC. UVM. |
|  | 1 (23 cancers) | ZNF695 | ACC. AML. BLCA. BRCA. COAD. ESAD. ESCA. ESCC. GBM. HNSC. KICH. KIRC. KIRP. LIHC. LUAD. OSCC. PAAD. PCPG. PRAD. SARC. SKCM. STAD. UCEC. |
|  | 1 (23 cancers) | NUF2 | ACC. AML. BRCA. CHOL. GBM. KICH. KIRC. KIRP. LGG. LIHC. LUAD. MESO. OSCC. OV. PAAD. PCPG. PRAD. SARC. SKCM. THCA. THYM. UCEC. UVM. |
|  | 1 (23 cancers) | ESCO2 | ACC. AML. BRCA. COAD. DLBC. GBM. KICH. KIRC. KIRP. LGG. LIHC. LUAD. MESO. OV. PAAD. PCPG. PRAD. SARC. SKCM. THCA. THYM. UCEC. UVM. |
|  | 1 (22 cancers) | PIMREG | ACC. AML. BLCA. BRCA. CHOL. ESAD. GBM. KICH. KIRC. KIRP. LGG. LIHC. LUAD. MESO. PAAD. PCPG. PRAD. SARC. TGCT. THCA. THYM. UCEC. |
|  | 1 (22 cancers) | KIF4A | ACC. AML. BLCA. BRCA. CHOL. ESAD. KICH. KIRC. KIRP. LGG. LIHC. LUAD. MESO. PAAD. PCPG. PRAD. SARC. STAD. TGCT. THCA. THYM. UCEC. |
|  | 1 (22 cancers) | CKAP2L | ACC. AML. BLCA. BRCA. CHOL. GBM. KICH. KIRC. KIRP. LGG. LIHC. LUAD. MESO. PAAD. PCPG. PRAD. SARC. SKCM. THCA. THYM. UCEC. UVM. |
|  | 1 (22 cancers) | KIF23 | ACC. AML. BLCA. BRCA. CHOL. GBM. KICH. KIRC. KIRP. LGG. LIHC. LUAD. MESO. PAAD. PCPG. PRAD. SARC. STAD. THCA. THYM. UCEC. UVM. |
|  | 1 (22 cancers) | CENPE | ACC. AML. BLCA. BRCA. CHOL. GBM. KICH. KIRC. KIRP. LGG. LIHC. LUAD. MESO. PAAD. PRAD. SARC. SKCM. STAD. THCA. THYM. UCEC. UVM. |
|  | 1 (22 cancers) | ASPM | ACC. AML. BLCA. BRCA. CHOL. GBM. KICH. KIRC. KIRP. LGG. LIHC. LUAD. PAAD. PCPG. PRAD. SARC. SKCM. STAD. THCA. THYM. UCEC. UVM. |
|  | 1 (21 cancers) | FAM72B | ACC. AML. BLCA. BRCA. CHOL. DLBC. ESAD. KICH. KIRP. LGG. LIHC. LUAD. MESO. OSCC. PAAD. PRAD. SARC. THCA. THYM. UCEC. UVM. |
|  | 1 (21 cancers) | CEP55 | ACC. AML. BLCA. BRCA. CHOL. GBM. KICH. KIRC. KIRP. LGG. LIHC. LUAD. MESO. PAAD. PCPG. PRAD. SARC. THCA. THYM. UCEC. UVM. |
|  | 1 (21 cancers) | TEX15 | ACC. AML. BLCA. BRCA. ESAD. ESCA. ESCC. HNSC. KICH. KIRP. LIHC. LUAD. LUSC.MESO. OSCC. PAAD. PRAD. SARC. SKCM. UCEC. UVM. |
|  | 1 (21 cancers) | KIF20A | ACC. AML. BRCA. CHOL. DLBC. ESAD. GBM. KICH. KIRC. KIRP. LGG. LIHC. LUAD. MESO. PAAD. PCPG. PRAD. SARC. THCA. THYM. UVM. |
|  | 1 (21 cancers) | BUB1 | ACC. AML. BRCA. CHOL. GBM. KICH. KIRC. KIRP. LGG. LIHC. LUAD. MESO. PAAD. PCPG. PRAD. SARC. STAD. THCA. THYM. UCEC. UVM. |
|  | 1 (21 cancers) | NEIL3 | ACC. BLCA. BRCA. CHOL. GBM. KICH. KIRC. KIRP. LGG. LIHC. LUAD. MESO. OV. PAAD. PCPG. PRAD. SARC. THCA. THYM. UCEC. UVM. |
|  | 1 (20 cancers) | KIF18B | ACC. AML. BLCA. BRCA. CHOL. ESAD. GBM. KICH. KIRC. KIRP. LGG. LIHC. LUAD. MESO. PAAD. PRAD. SARC. TGCT. THCA. THYM. |
|  | 1 (20 cancers) | NDC80 | ACC. AML. BLCA. BRCA. CHOL. GBM. KICH. KIRP. LGG. LIHC. LUAD. MESO. PAAD. PCPG. SARC. SKCM. THCA. THYM. UCEC.UVM. |
|  | 1 (20 cancers) | CDCA2 | ACC. AML. BLCA. BRCA. CHOL. GBM. KICH. KIRP. LGG. LIHC. LUAD. MESO. PAAD. PCPG. PRAD. SARC. STAD. THCA. THYM. UCEC. |
|  | 1 (20 cancers) | KNL1 | ACC. AML. BLCA. BRCA. CHOL. GBM. KIRC. KIRP. LGG. LUAD. MESO. PAAD. PCPG. PRAD. SARC. SKCM. THCA. THYM. UCEC. UVM. |
|  | 1 (20 cancers) | MELK | ACC. AML. BLCA. BRCA. GBM. KICH. KIRC. KIRP. LGG. LIHC. LUAD. MESO. PAAD. PCPG. PRAD. SARC. THCA. THYM. UCEC. UVM. |
|  | 1 (20 cancers) | CDK1 | ACC. AML. BRCA. CHOL. GBM. KICH. KIRC. KIRP. LGG. LIHC. LUAD. MESO. PAAD. PCPG. PRAD. SARC. TGCT. THYM. UCEC. UVM. |
|  | 1 (20 cancers) | CENPA | ACC. AML. BRCA. CHOL. GBM. KICH. KIRC. KIRP. LGG. LIHC. LUAD. MESO. PAAD. PCPG. PRAD. SARC. THCA. THYM. UCEC. UVM. |
|  | 1 (20 cancers) | KIF15 | ACC. AML. BRCA. CHOL. GBM. KICH. KIRC. KIRP. LGG. LIHC. LUAD. MESO. PAAD. PRAD. SARC. SKCM. THCA. THYM. UCEC. UVM. |
|  | 1 (20 cancers) | DEPDC1B | ACC. AML. BRCA. GBM. KICH. KIRC. KIRP. LGG. LIHC. LUAD. MESO. PAAD. PCPG. PRAD. SARC. SKCM. TGCT. THYM. UCEC. UVM. |
|  | 1 (20 cancers) | FOXM1 | ACC. BLCA. BRCA. CHOL. DLBC. KICH. KIRC. KIRP. LGG. LIHC. LUAD. MESO. PAAD. PCPG. PRAD. SARC. TGCT. THCA. THYM. UCEC. |
|  | 1 (20 cancers) | ERCC6L | ACC. BRCA. CHOL. ESAD. GBM. KICH. KIRC. KIRP. LGG. LIHC. LUAD. MESO. PAAD. PCPG. PRAD. SARC. SKCM. THYM. UCEC. UVM. |
|  | 1 (20 cancers) | RRM2 | ACC. BRCA. CHOL. GBM. KICH. KIRC. KIRP. LGG. LIHC. LUAD. MESO. PAAD. PCPG. PRAD. SARC. TGCT. THCA. THYM. UCEC. UVM. |
|  | 1 (19 cancers) | SEPTIN14P12 | ACC. AML. BLCA. BRCA. CHOL. COAD. GBM. KIRC. LGG. LIHC. LUAD. MESO. OSCC. OV. PAAD. SARC. SKCM. THYM. UCEC. |
|  | 1 (19 cancers) | CDC20 | ACC. AML. BLCA. BRCA. CHOL. GBM. KICH. KIRC. KIRP. LGG. LIHC. LUAD. MESO. PAAD. PCPG. PRAD. SARC. TGCT. THYM. |
|  | 1 (19 cancers) | PLK1 | ACC. AML. BLCA. BRCA. CHOL. GBM. KICH. KIRC. KIRP. LGG. LIHC. LUAD. MESO. PAAD. PRAD. SARC. TGCT. THCA. THYM. |
|  | 1 (19 cancers) | TPX2 | ACC. AML. BLCA. BRCA. CHOL. KICH. KIRC. KIRP. LGG. LIHC. LUAD. MESO. PAAD. PCPG. PRAD. SARC. THCA. THYM. UCEC. |
|  | 2 (19 cancers) | MKI67 NCAPH | ACC. AML. BLCA. BRCA. GBM. KICH. KIRC. KIRP. LGG. LIHC. LUAD. MESO. PAAD. PCPG. PRAD. SARC. THCA. THYM. UCEC. |
|  | 1 (19 cancers) | E2F8 | ACC. AML. BRCA. CHOL. DLBC. KIRC. KIRP. LGG. LIHC. LUAD. PAAD. PCPG. PRAD. SARC. SKCM. THCA. THYM. UCEC. UVM. |
|  | 1 (19 cancers) | GTSE1 | ACC. AML. BRCA. CHOL. GBM. KICH. KIRC. KIRP. LGG. LIHC. LUAD. MESO. PAAD. PCPG. PRAD. SARC. THCA. THYM. UCEC. |
|  | 1 (19 cancers) | H3C2 | ACC. BLCA. BRCA. COAD. ESAD. GBM. KICH. KIRC. KIRP. LGG. LUAD. MESO. PAAD. PCPG. PRAD. SARC. THCA. THYM. UCEC. |
|  | 1 (19 cancers) | LIN28B | ACC. BLCA. BRCA. ESAD. ESCA. ESCC. HNSC. KIRC. KIRP. LIHC. LUAD. LUSC. MESO.OSCC  READ. SARC. SKCM. STAD. TGCT. |
|  | 1 (19 cancers) | KIF18A | ACC. BLCA. BRCA. GBM. KICH. KIRC. KIRP. LGG. LIHC. LUAD. MESO. PAAD. PCPG. PRAD. SARC. SKCM. THYM. UCEC. UVM. |
|  | 1 (19 cancers) | SGO1 | ACC. BRCA. CHOL. GBM. KICH. KIRC. KIRP. LGG. LIHC. LUAD. MESO. PAAD. PCPG. PRAD. SARC. SKCM. THCA. THYM. UCEC. |
|  | 1 (19 cancers) | AC104073.4 | AML. BLCA. BRCA. CHOL. ESAD. ESCA. ESCC. GBM. HNSC. KIRC. KIRP. LGG. LIHC. LUAD. MESO. OSCC. OV. SKCM. THYM. |
|  | 2 (19 cancers) | MAGEA6 MAGEA3 | BLCA. BRCA. CESC. ESAD. ESCA. ESCC. KIRC. KIRP. LGG. LIHC. LUAD. MESO. OV. PAAD. PCPG. PRAD. READ. SARC. UCEC. |
|  | 1 (18 cancers) | CDCA8 | ACC. AML. BLCA. BRCA. CHOL. GBM. KICH. KIRC. KIRP. LGG. LIHC. LUAD. MESO. PAAD. PCPG. SARC. THYM. UCEC. |
|  | 1 (18 cancers) | TCAM1P | ACC. AML. BLCA. BRCA. ESAD. HNSC. KICH. KIRC. KIRP. LIHC. LUAD. LUSC. OSCC. PAAD. PCPG. SARC. STAD. UCEC. |
|  | 1 (18 cancers) | CDKN3 | ACC. AML. BLCA. BRCA. GBM. KICH. KIRC. KIRP. LGG. LIHC. LUAD. MESO. PAAD. PRAD. SARC. THCA. THYM. UCEC. |
|  | 1 (18 cancers) | MAGEB2 | ACC. AML BLCA. COAD. ESAD. ESCA. ESCC. KIRC. LIHC. LUAD. LUSC. MESO. OV. PAAD. SARC. STAD. UCEC. UCS. |
|  | 1 (18 cancers) | AC015849.5 | ACC. AML. BRCA. CHOL. DLBC. GBM. HNSC. KIRC. KIRP. LGG. LUAD. MESO. OSCC. PCPG. PRAD. SARC. THYM. UCEC. |
|  | 1 (18 cancers) | BIRC5 | ACC. AML. BRCA. CHOL. GBM. KICH. KIRC. KIRP. LGG. LIHC. LUAD. MESO. OV. PAAD. PCPG. PRAD. SARC. THCA. |
|  | 4 (18 cancers) | CDC25C HJURP SKA1 UBE2C | ACC. AML. BRCA. CHOL. GBM. KICH. KIRC. KIRP. LGG. LIHC. LUAD. MESO. PAAD. PCPG. PRAD. SARC. THCA. THYM. |
|  | 2 (18 cancers) | CENPI EXO1 | ACC. BLCA. BRCA. CHOL. GBM. KICH. KIRC. KIRP. LGG. LIHC. LUAD. MESO. PAAD. PCPG. PRAD. SARC. THYM. UCEC. |
|  | 1 (18 cancers) | ARHGAP11A | ACC. BLCA. BRCA. CHOL. KICH. KIRC. KIRP. LGG. LIHC. LUAD. MESO. PCPG. PRAD. SARC. SKCM. THYM. UCEC. UVM. |
|  | 1 (18 cancers) | FAM111B | ACC. BLCA. BRCA. DLBC. GBM. HNSC. KICH. KIRP. LGG. LIHC. LUAD. MESO. PCPG. PRAD. SARC. TGCT. THYM. UVM. |
|  | 1 (18 cancers) | HASPIN | ACC. BLCA. BRCA. DLBC. GBM. KICH. KIRC. KIRP. LGG. LIHC. LUAD. MESO. PCPG. PRAD. SARC. THCA. THYM. UCEC. |
|  | 1 (18 cancers) | E2F7 | ACC. BLCA. BRCA. DLBC. HNSC. KICH. KIRC. KIRP. LGG. LIHC. LUAD. MESO. OSCC. PAAD. PCPG. PRAD. SARC. THYM. |
|  | 1 (18 cancers) | AURKB | ACC. BRCA. CHOL. GBM. KICH. KIRC. KIRP. LGG. LIHC. LUAD. MESO. PAAD. PCPG. PRAD. SARC. TGCT. THCA. THYM. |
|  | 1 (18 cancers) | POLQ | ACC. BRCA. CHOL. KICH. KIRC. KIRP. LGG. LIHC. LUAD. MESO. PAAD. PCPG. PRAD. SARC. SKCM. THCA. THYM. UCEC. |
|  | 1 (18 cancers) | GFY | BLCA. BRCA. ESAD. ESCA. ESCC. HNSC. KICH. KIRC. LIHC. LUAD. LUSC. MESO. OV. PAAD. PCPG. READ. STAD. UCEC. |
|  | 1 (17 cancers) | FAM72A | ACC. AML. BLCA. BRCA. CHOL. DLBC. ESAD. KICH. KIRP. LIHC. LUAD. MESO. PRAD. SARC. THYM. UCEC. UVM. |
|  | 1 (17 cancers) | DIAPH3 | ACC. AML. BLCA. BRCA. DLBC. LIHC. LUAD. PAAD. PCPG. PRAD. SARC. SKCM. TGCT. THCA. THYM. UCEC. UVM. |
|  | 1 (17 cancers) | TMSB15A | ACC. AML BLCA. BRCA. GBM. HNSC. KICH. KIRP. LGG. LUAD. LUSC. OSCC. OV. SARC. SKCM. THYM. UCEC. |
|  | 1 (17 cancers) | FBXO43 | ACC. AML. BLCA. CHOL. GBM. HNSC. KICH. KIRC. LIHC. LUAD. MESO. OSCC. OV. SARC. SKCM. THYM. UVM. |
|  | 1 (17 cancers) | CCNB2 | ACC. AML. BRCA. CHOL. GBM. KICH. KIRC. KIRP. LGG. LIHC. LUAD. MESO. PAAD. PCPG. SARC. THYM. UVM. |
|  | 1 (17 cancers) | KIF2C | ACC. AML. BRCA. CHOL. GBM. KICH. KIRC. KIRP. LGG. LIHC. LUAD. MESO. PAAD. PRAD. SARC. THYM. UCEC. |
|  | 1 (17 cancers) | FAM230C | ACC. BLCA. BRCA. CESC. ESAD. ESCA. ESCC. KIRP. LGG. LIHC. LUAD. LUSC. MESO. PCPG. STAD. UCEC. UCS. |
|  | 1 (17 cancers) | PRAME | ACC. BLCA. BRCA. ESAD. ESCA. ESCC. GBM. KICH. KIRC. LGG. LIHC. LUAD. MESO. PAAD. PRAD. STAD. THCA. |
|  | 1 (17 cancers) | MCM10 | ACC. BLCA. BRCA. GBM. KICH. KIRC. KIRP. LGG. LIHC. LUAD. MESO. PAAD. PRAD. SARC. THYM. UCEC. UVM. |
|  | 1 (17 cancers) | RAD51AP1 | ACC. BLCA. BRCA. KICH. KIRC. LGG. LIHC. LUAD. MESO. OSCC. PAAD. SARC. SKCM. TGCT. THYM. UCEC. UVM. |
|  | 3 (17 cancers) | SKA3 TROAP KIFC1 | ACC. BRCA. CHOL. GBM. KICH. KIRC. KIRP. LGG. LIHC. LUAD. MESO. PAAD. PCPG. PRAD. SARC. THCA. THYM. |
|  | 1 (17 cancers) | ZNF670-ZNF695 | ACC. BRCA. ESAD. ESCA. ESCC. GBM. HNSC. KICH. KIRC. KIRP. LGG. LIHC. LUAD. OSCC. SKCM. THYM. UVM. |
|  | 1 (17 cancers) | KIF11 | ACC. BRCA. GBM. KICH. KIRC. KIRP. LGG. LIHC. LUAD. MESO. PAAD. PCPG. PRAD. SARC. THYM. UCEC. UVM. |
|  | 1 (17 cancers) | CENPK | ACC. DLBC. GBM. HNSC. KICH. KIRP. LGG. LIHC. LUAD. MESO. OSCC. PAAD. PRAD. SARC. SKCM. THYM. UVM. |
|  | 1 (17 cancers) | RNA5SP323 | AML. BLCA. BRCA. COAD. DLBC. GBM. KIRC. LGG. LUAD. MESO. PAAD. SARC. SKCM. STAD. THYM. UCEC. UCS. |
|  | 1 (16 cancers) | FAM72C | ACC. AML. BRCA. CHOL. ESAD. GBM. KICH. KIRC. KIRP. LGG. LIHC. LUAD. MESO. PRAD. SARC. THYM. |
|  | 1 (16 cancers) | AC079841.2 | ACC. AML. BRCA. COAD. ESCA. ESCC. GBM. LIHC. LUAD. MESO. SARC. SKCM. STAD. THYM. UCEC. UCS. |
|  | 1 (16 cancers) | PRDM13 | ACC. BLCA. BRCA. CESC. HNSC. KIRP. LGG. LIHC. LUAD. MESO. OSCC. OV. SARC. STAD. THYM. UCS. |
|  | 1 (16 cancers) | ULBP1 | ACC. BLCA. BRCA. CHOL. ESAD. KICH. KIRP. LGG. LIHC. OSCC. OV. STAD. TGCT. UCEC. UCS. UVM. |
|  | 1 (16 cancers) | MAD2L1 | ACC. BLCA. BRCA. CHOL. GBM. LGG. LIHC. LUAD. MESO. OV. PAAD. SARC. SKCM. TGCT. THYM. UCEC. |
|  | 1 (16 cancers) | CLSPN | ACC. BLCA. BRCA. CHOL. KICH. KIRP. LGG. LIHC. LUAD. MESO. PAAD. PRAD. SARC. THYM. UCEC. UVM. |
|  | 1 (16 cancers) | IGF2BP3 | ACC. BLCA. BRCA. COAD. ESAD. KIRC. KIRP. LGG. LIHC. LUAD. MESO. PAAD. READ. TGCT. UCEC. UVM. |
|  | 1 (16 cancers) | IGF2BP1 | ACC. BLCA. BRCA. ESAD. ESCA. ESCC. GBM. LUAD. MESO. PAAD. READ. SARC. SKCM. STAD. THCA. UCEC. |
|  | 1 (16 cancers) | PASD1 | ACC. BLCA. BRCA. ESAD. ESCA. ESCC. KIRC. KIRP. LGG. LIHC. LUAD. LUSC. SARC. SKCM. STAD. UCEC. |
|  | 1 (16 cancers) | LINC00973 | ACC. BLCA. BRCA. ESAD. ESCA. ESCC. KIRC. KIRP. LUAD. MESO. OSCC. PAAD. READ. SKCM. STAD. THCA. |
|  | 1 (16 cancers) | ORC1 | ACC. BLCA. BRCA. GBM. KICH. KIRC. KIRP. LGG. LIHC. LUAD. MESO. PAAD. SARC. TGCT. THYM. UCEC. |
|  | 1 (16 cancers) | MAGEA12 | ACC. BRCA. CESC. ESAD. ESCA. ESCC. KIRP. LIHC. LUAD. LUSC. MESO. PRAD. READ. SARC. STAD. UCEC. |
|  | 1 (16 cancers) | DDIAS | ACC. BRCA. CHOL. KICH. KIRC. KIRP. LGG. LIHC. LUAD. MESO. PRAD. SARC. SKCM. THYM. UCEC. UVM. |
|  | 1 (16 cancers) | SHCBP1 | ACC. BRCA. KICH. KIRC. KIRP. LGG. LIHC. LUAD. MESO. PAAD. PCPG. PRAD. SARC. THCA. THYM. UVM. |
|  | 1 (16 cancers) | LRRC37A9P | AML. BRCA. CHOL. DLBC. GBM. HNSC. KIRC. KIRP. LGG. LUAD. PCPG. PRAD. SARC. THYM. UCEC. UCS. |
|  | 1 (16 cancers) | LINC01446 | BLCA. BRCA. COAD. ESAD. ESCA. ESCC. LIHC. LUAD. LUSC. OV. PRAD. READ. STAD. THCA. THYM. UCEC. |
|  | 1 (16 cancers) | MAGEA10 | BLCA. BRCA. ESAD. ESCA. ESCC. HNSC. KIRC. LIHC. LUAD. OSCC. OV. READ. SKCM. STAD. THYM. UCEC. |
|  | 1 (16 cancers) | AL359894.1 | BRCA. CESC. ESCA. ESCC. KIRC. KIRP. LIHC. LUAD. LUSC. OV. PCPG. READ. SARC. STAD. UCEC. UCS. |
|  | 1 (16 cancers) | DCAF4L2 | BRCA. ESAD. ESCA. ESCC. GBM. HNSC. LGG. LIHC. LUAD. OSCC. OV. PRAD. SARC. STAD. UCEC. UCS. |
| **Low expression genes** | 1 (17 cancers) | C7 | BLCA. BRCA. CHOL. COAD. DLBC. ESAD. ESCA. ESCC. LIHC. LUAD. LUSC. MESO. PAAD.  READ. SARC. STAD. THYM. |
|  | 1 (16 cancers) | MAL | ACC. BLCA. CESC. CHOL. DLBC. ESAD. ESCA. ESCC. HNSC. KIRC. OSCC. SARC. SKCM. STAD. TGCT. UVM. |
|  | 1 (16 cancers) | IGHD | ACC. BLCA. CHOL. DLBC. ESAD. ESCA. ESCC. LUSC. MESO. READ. SARC. SKCM. STAD. THYM. UCEC. UVM. |
|  | 1 (16 cancers) | PTGDS | ACC. BLCA. CHOL. DLBC. ESAD. LUAD. LUSC. PCPG. PRAD. READ. SARC. SKCM. STAD. TGCT. UCEC. UVM. |
|  | 1 (16 cancers) | ADH1B | ACC. BLCA. ESAD. ESCA. ESCC. LIHC. LUAD. LUSC. MESO. OV. SARC. STAD. THYM. UCEC. UCS. UVM. |
|  | 1 (16 cancers) | CCN5 | BLCA. BRCA. COAD. ESAD. ESCA. ESCC. LIHC. MESO. OSCC. PRAD. READ. SARC. STAD. TGCT. THYM. UVM. |

**Table S4. The no. of transcription factors upstream of DEPDC1 predicted by eight databases.**

| **Database** | **The number of predicted TFs upstream of DEPDC1** |
| --- | --- |
| ChIP Atlas | 126 |
| CistromeDB Toolkit | 101 |
| ENCODE | 59 |
| GTRD | 126 |
| Gene card | 123 |
| HumanTFDB | 126 |
| JASPAR | 326 |
| hTFtarget | 116 |
| **Overall number of unique TFs** | **703** |

**Table S5. The predicted transcription factors upstream of DEPDC1.**

| **Database** | **The no. of TFs** | **The potential TFs upstream of DEPDC1** |
| --- | --- | --- |
| ChIP Atlas CistromeDB Toolkit  ENCODE  GTRD  Gene card  HumanTFDB hTFtarget | 1 | FOXM1 |
| ChIP Atlas CistromeDB Toolkit  ENCODE  GTRD HumanTFDB  JASPAR  hTFtarget | 1 | GATA1 |
| ChIP Atlas CistromeDB Toolkit  ENCODE  GTRD  Gene card  hTFtarget | 3 | ATF3 HDAC1 E2F4 |
| ChIP Atlas CistromeDB Toolkit ENCODE  GTRD  HumanTFDB  hTFtarget | 2 | EP300 CTCF |
| ChIP Atlas  CistromeDB Toolkit  ENCODE  GTRD  JASPAR  hTFtarget | 1 | FOS |
| ChIP Atlas CistromeDB Toolkit  GTRD  HumanTFDB JASPAR  hTFtarget | 1 | FOXA1 |
| ChIP Atlas  ENCODE  GTRD  Gene card  HumanTFDB hTFtarget | 3 | CREB1 HDAC2 ELF1 |
| ChIP Atlas  ENCODE  GTRD  HumanTFDB JASPAR  hTFtarget | 1 | E2F6 |
| ChIP Atlas  CistromeDB Toolkit  GTRD  HumanTFDB  hTFtarget | 4 | CDK9 BRD4 ESR1 AR |
| ChIP Atlas  CistromeDB Toolkit GTRD  JASPAR  hTFtarget | 1 | CTCFL |
| ChIP Atlas  ENCODE  GTRD  Gene card  hTFtarget | 1 | BHLHE40 |
| ChIP Atlas  ENCODE  GTRD  HumanTFDB  hTFtarget | 1 | ETS1 |
| ChIP Atlas  ENCODE  GTRD  JASPAR  hTFtarget | 1 | GATA2 |
| ChIP Atlas  ENCODE  HumanTFDB  JASPAR  hTFtarget | 1 | EGR1 |
| ChIP Atlas  GTRD  Gene card HumanTFDB JASPAR | 1 | IKZF1 |
| CistromeDB Toolkit  ENCODE  GTRD  Gene card  hTFtarget | 1 | CEBPB |
| CistromeDB Toolkit  ENCODE  GTRD  HumanTFDB  hTFtarget | 1 | CBX3 |
| GTRD  Gene card HumanTFDB JASPAR  hTFtarget | 1 | KLF1 |
| ChIP Atlas  CistromeDB Toolkit  GTRD  HumanTFDB | 1 | CDK8 |
| ChIP Atlas  CistromeDB Toolkit  HumanTFDB hTFtarget | 1 | MED1 |
| ChIP Atlas  ENCODE  GTRD  Gene card | 1 | GTF2F1 |
| ChIP Atlas  ENCODE GTRD  hTFtarget | 1 | FLI1 |
| ChIP Atlas  ENCODE  GTRD  HumanTFDB | 1 | GATA3 |
| ChIP Atlas  GTRD  Gene card HumanTFDB | 1 | BCL6 |
| ChIP Atlas  GTRD  HumanTFDB  hTFtarget | 3 | ERG FOXA2 CREBBP |
| ChIP Atlas  GTRD  JASPAR  hTFtarget | 1 | E2F1 |
| CistromeDB Toolkit  ENCODE  HumanTFDB  hTFtarget | 2 | KDM5B NANOG |
| CistromeDB Toolkit ENCODE  JASPAR  hTFtarget | 1 | NFE2 |
| CistromeDB Toolkit Gene card HumanTFDB hTFtarget | 3 | USF2 TRIM28 POLR2A |
| ENCODE  GTRD  Gene card  JASPAR | 1 | BACH1 |
| ENCODE  Gene card  HumanTFDB  hTFtarget | 1 | MYC |
| Gene card HumanTFDB  JASPAR  hTFtarget | 1 | SP1 |
| GTRD  HumanTFDB  JASPAR  hTFtarget | 1 | E2F8 |
| ChIP Atlas CistromeDB Toolkit  ENCODE | 1 | CHD1 |
| ChIP Atlas  CistromeDB Toolkit GTRD | 1 | EHMT2 |
| ChIP Atlas  CistromeDB Toolkit  hTFtarget | 1 | CBFB |
| ChIP Atlas  ENCODE  GTRD | 3 | CHD2 EZH2 CCNT2 |
| ChIP Atlas  ENCODE  JASPAR | 1 | FOXP2 |
| ChIP Atlas  GTRD  Gene card | 8 | GLIS1 ATF1 DMAP1 GATAD1 HBP1 CBX1 ARID4B GABPB1 |
| ChIP Atlas  Gene card HumanTFDB | 1 | GMEB1 |
| ChIP Atlas  GTRD  hTFtarget | 2 | KMT2A FOXP1 |
| ChIP Atlas  GTRD  HumanTFDB | 3 | BRD7 BRD2 BRD3 |
| ChIP Atlas  GTRD  JASPAR | 2 | FOSL2 KLF10 |
| ChIP Atlas  HumanTFDB  JASPAR | 2 | HIF1A FOXK1 |
| CistromeDB Toolkit ENCODE  HumanTFDB | 1 | KDM1A |
| CistromeDB Toolkit Gene card  hTFtarget | 4 | PHF8 YY1 SIN3A ZFX |
| CistromeDB Toolkit Gene card HumanTFDB | 1 | SMARCA4 |
| CistromeDB Toolkit GTRD  HumanTFDB | 1 | IRF4 |
| CistromeDB Toolkit  HumanTFDB hTFtarget | 3 | SUZ12 RELA RUNX1 |
| ENCODE  Gene card  hTFtarget | 2 | MAX NRF1 |
| ENCODE  Gene card  HumanTFDB | 1 | HCFC1 |
| ENCODE  Gene card  JASPAR | 1 | ELK1 |
| ENCODE  GTRD  HumanTFDB | 1 | BRCA1 |
| ENCODE HumanTFDB hTFtarget | 1 | IRF1 |
| ENCODE  JASPAR  hTFtarget | 1 | NFYA |
| ENCODE  HumanTFDB JASPAR | 1 | IRF3 |
| Gene card HumanTFDB  hTFtarget | 5 | TAF1 REST TCF12 UBTF TBP |
| Gene card HumanTFDB JASPAR | 1 | SP2 |
| GTRD  HumanTFDB hTFtarget | 1 | ARNT |
| GTRD  JASPAR  hTFtarget | 2 | GRHL2 GMEB2 |
| GTRD  HumanTFDB JASPAR | 2 | EGR2 GABPA |
| HumanTFDB JASPAR  hTFtarget | 1 | POU5F1 |
| ChIP Atlas  Gene card | 5 | KLF11 DPF2 ASH2L DEAF1 CHAMP1 |
| ChIP Atlas  GTRD | 19 | CHD8 BRD9 AFF1 DDIT3 CDKN1B CBFA2T2 AFF4 DRAP1 GTF3C5 BRPF3 ETV5 HNRNPLL ASF1A CRTC2 CNOT3 E2F5 GATAD2B BCL11B ARID2 |
| ChIP Atlas  hTFtarget | 1 | BMI1 |
| ChIP Atlas HumanTFDB | 4 | HIVEP1 ASCL2 BCL3 BCOR |
| ChIP Atlas  JASPAR | 1 | FOSL1 |
| CistromeDB Toolkit Gene card | 5 | ZBTB48 SUPT5H RAD51 ZSCAN29 SREBF1 |
| CistromeDB Toolkit GTRD | 2 | AGO1 CHD4 |
| CistromeDB Toolkit hTFtarget | 8 | MITF SUMO2 SMC3 TAL1 ZNF711 RAD21 ZBTB7A RBL2 |
| CistromeDB Toolkit HumanTFDB | 4 | SMAD3 T RNF2 SRF |
| CistromeDB Toolkit JASPAR | 4 | TCF7L1 ZEB1 SNAI2 ZNF682 |
| ENCODE  GTRD | 2 | CHD7 H2AFZ |
| ENCODE  hTFtarget | 6 | KDM4A MXI1 MAFK JUND NFYB MAZ |
| ENCODE HumanTFDB | 1 | HDAC6 |
| GTRD  Gene card | 4 | HOMEZ LARP7 EP400 CREM |
| Gene card  hTFtarget | 3 | PML TAF7 MNT |
| Gene card HumanTFDB | 2 | SP3 SMAD4 |
| Gene card  JASPAR | 7 | ZNF610 FOXK2 MGA MEIS2 ATF2 IRF2 NFYC |
| GTRD  hTFtarget | 2 | CDX2 CDK7 |
| GTRD  HumanTFDB | 4 | BCL11A GRHL3 EPAS1 GTF3C2 |
| GTRD  JASPAR | 5 | GATA6 ELF3 CEBPA CEBPD GATA4 |
| HumanTFDB hTFtarget | 8 | SPI1 TAF3 LMO2 STAT3 NFAT5 HOXA6 NOTCH1 SOX2 |
| JASPAR  hTFtarget | 7 | FOXD2 TFDP1 TFAP2C USF1 MYOD1 SP4 MAFF |
| HumanTFDB JASPAR | 7 | TCF4 PBX1 ZNF143 IRF7 HOXA10 FOXH1 MYB |
| ChIP Atlas | 38 | FOXP4 AHDC1 KMT2D CDYL2 BCORL1 JADE3 CIC DZIP1 CDT1 CPSF6 FOXL2 CHD6 ARID5B GLIS2 HOXB13 HSF1 CC2D1A ARID4A ELAVL1 HAND2 GTF2E2 AKAP8 ASPSCR1 CPSF4 ARHGAP35 HES4 ASCL1 CXXC5 ETV6 INTS12 EEA1 FOXO1 DOT1L EMSY BRD1 CPSF2 HMGB1 GZF1 |
| CistromeDB Toolkit | 41 | IRAK1 ZNF84 NUP98 HOTAIR MECP2 ZNF430 ZNF793 NELFA NKX3-1 ZNF786 SETDB1 ZNF284 TET2 SP140 SCML2 NIPBL PHF2 ZNF674 NPAT DPF1 MYCN MXD3 ZNF273 KRAB CDK2 H2AZ PR GRIP1 TET1 ZNF561 KDM2B MORC2 NEUROD1 SMAD2/3 HMBOX1 LMNA PIAS1 POLR2M INTS3 ZBTB17 ZNF618 |
| ENCODE | 10 | MTA3 BCLAF1 NELFE HMGN3 KDM5A GTF2B MYBL2 NFIC JUN NR2F2 |
| Gene card | 54 | SP7 ZNF664 ZNF189 TAF9B HLTF ZNF843 ZNF580 ZSCAN5A SAP130 IKZF5 L3MBTL2 MLLT1 ZNF48 HIC1 PHF20 CBLL2 ZNF354B KLF16 TFAP4 RBPJ ZFP37 MXD4 ZNF501 NKRF SKI PATZ1 PRDM10 PRDM4 OSR2 TARDBP CTBP1 ZHX1 TGIF2 ZBTB26 SKIL PKNOX1 ZNF2 KAT8 KMT2B ZBTB5 ZXDB TOE1 ZNF341 KDM3A MBD2 ATF4 NCOA6 ZBTB8A SIN3B ZBTB40 ZNF335 SMAD5 PHF21A RLF |
| GTRD | 26 | ATRX EED DCP1A FIP1L1 FUS CXXC1 GCM2 GATAD2A AHR ERCC6 HDAC3 HDGFL3 CCND2 CXXC4 EBF1 ARRB1 ELL2 CENPA DEK ERCC3 HMGA1 ME1 HNRNPL EGR3 ARNTL CBX2 |
| hTFtarget | 18 | SPIB EZH1 KDM4C SMAD1 STAG1 SAP30 SMC1A RB1 HEY1 XBP1 PGR NR3C1 ZNF263 TCF3 WDR5 RBBP5 E2F3 PAX5 |
| HumanTFDB | 37 | VDR DDX5 HIVEP2 IRX4 NFATC1 OTX2 SIX5 ZNF274 POU1F1 WHSC1 THAP11 ZBTB33 PURA PPARD/PPARG IRF5 NCOR1 ELSPBP1 DMRT2 TP73 STAT5A SALL4 KLF4 TP53 MBD3 GTF2I ZNF384 ETV4 NFKB1 ZBTB7B P50:RELA-P65 ZNF250 FOXO3 SMAD2/SMAD3 EOMES PBX2 STAT1 BTAF1 |
| JASPAR | 267 | BARHL2 ZNF684 Foxl2 Elf5 RXRG NR1H4::RXRA DBP VEZF1 ETV5::FOXI1 Stat2 ZNF701 ZBTB12 TGIF2LY POU4F2 Runx1 RFX5 RHOXF1 ZNF528 GSC UNCX ERF::FIGLA ARNT::HIF1A TBX4 Bcl11B Wt1 SOX4 HOXA1 NR4A2 RORA PAX4 NFIA NR2F1 Prdm15 Dlx3 INSM1 Tfcp2l1 KLF7 FLI1::DRGX RUNX3 SOX21 FOXN3 ZNF454 NFATC4 POU3F4 POU3F1 LIN54 Hic1 ETV3 GSX2 FEV ETV5::DRGX FOXO4 Hoxa13 PRDM9 TBX5 FOXO1::FLI1 FOXP3 Gli1 POU4F3 NRL ZNF257 Arid3b Zfx Npas4 ZNF317 NR4A1 Dlx4 Zic2 FOXC1 OSR1 PLAG1 Pax7 Msx3 FOXO1::ELK1 ZNF211 ZNF449 TEAD3 NKX6-1 FOSL1::JUNB ZNF460 KLF17 THRB MEF2A EN1 BARX2 Six4 SOX8 FOXI1 GSC2 Ascl2 FOXB1 Ddit3::Cebpa Plagl1 ERF::FOXO1 Thap11 ZBTB18 HES7 BARHL1 POU4F1 POU6F1 Znf423 Foxj2 NKX6-2 Hmx3 HOXB6 FOSB::JUNB Shox2 IRF6 RAX Sox11 Gli2 HOXA4 ETV2::DRGX NR2C2 FOXO6 MZF1 VSX1 KLF14 TBX20 Nkx2-1 OTX1 HOXA9 GFI1 FOS::JUN TFAP2A ETV2 PHOX2B RUNX2 ERF Lhx3 Sox3 BATF3 ZNF708 PRRX1 DLX6 THRA Spi1 Hmx2 FOXO1::ELK3 MAF::NFE2 TFEC HOXD3 TBX21 NR2C1 NEUROG1 POU2F3 PITX2 ARGFX FOXO1::ELF1 RORB Foxf1 PITX1 TEAD2 Gmeb1 FOXL1 Smad4 ZNF417 MAFG::NFE2L1 Foxo3 SOX12 GRHL1 RELB ZNF281 HNF1B ZNF680 DMRTC2 Vdr ZNF768 Mafb SIX2 Irf1 Nobox PHOX2A VSX2 SOX10 ZBED2 CREB3 Dux HINFP RARA::RXRA Lhx4 Zic1::Zic2 VAX1 Stat5b PITX3 Lhx8 Rbpjl HOXD12::ELK1 FOSL2::JUND LMX1B DMRTA1 SCRT2 GCM1 TCFL5 SIX1 ZKSCAN3 TBX1 SP8 Zic3 SCRT1 NKX6-3 ZNF740 FOXD1 FLI1::FOXI1 KLF12 TFCP2 OLIG1 ETV5::HOXA2 OLIG3 TFAP4::ETV1 SP5 HOXD9 FOXF2 NFATC3 NR1D2 Nr2e1 ZNF354A JDP2 POU2F1 ZBTB7C GLI3 RFX7 Spz1 TRPS1 BNC2 FERD3L LHX2 MEIS3 Creb5 EVX1 mix-a DPRX POU3F2 SPIC Gfi1B ESR2 HOXA7 GBX2 ZNF214 Stat5a::Stat5b LHX9 POU2F2 TBX15 ZBTB6 Foxo1 Stat5a Arid3a SATB1 EVX2 ERF::FOXI1 BATF POU5F1B Nkx3-2 ELK4 BATF::JUN DMRTA2 ETV5::FOXO1 EMX1 TEAD4 GBX1 ZBTB14 Ahr::Arnt FOXG1 MEF2C ERF::HOXB13 SRY Gata3 |

**Table S6. The no. of predicted miRNAs targeting DEPDC1.**

| **miRNA databases** | **The number of miRNAs** |
| --- | --- |
| TargetScanHuman_8.0 | 630 |
| DIANA-TarBase v9.0 | 131 |
| DIANA-MicroT-CDS | 164 |
| StarBase | 72 |
| MiRDB | 132 |
| MiRWalk | 804 |
| **Overall number of unique elements** | **1354** |

**Table S7. The predicted miRNAs targeting DEPDC1.**

| **miRNA databases** | **total** | **The potential miRNAs silencing DEPDC1 expression** |
| --- | --- | --- |
| DIANA-MicroT-CDS  DIANA-TarBase v9.0 StarBase  TargetScanHuman_8.0 miRDB.txt miRWalk | 1 | hsa-miR-96-5p |
| DIANA-MicroT-CDS DIANA-TarBase v9.0 StarBase  TargetScanHuman_8.0  miRDB | 6 | hsa-miR-23a-3p hsa-miR-23b-3p hsa-miR-1271-5p hsa-miR-374a-5p hsa-miR-374b-5p hsa-miR-454-3p |
| DIANA-MicroT-CDS DIANA-TarBase v9.0 StarBase  TargetScanHuman_8.0 miRWalk | 2 | hsa-miR-19b-3p hsa-miR-182-5p |
| DIANA-MicroT-CDS  StarBase TargetScanHuman_8.0 miRDB  miRWalk | 4 | hsa-miR-506-3p hsa-miR-432-5p hsa-miR-3666 hsa-miR-4661-5p |
| DIANA-TarBase v9.0  StarBase  TargetScanHuman_8.0 miRDB  miRWalk | 3 | hsa-miR-301a-3p hsa-miR-130a-3p hsa-miR-130b-3p |
| DIANA-MicroT-CDS DIANA-TarBase v9.0 StarBase  miRDB | 1 | hsa-miR-124-3p |
| DIANA-MicroT-CDS  DIANA-TarBase v9.0 TargetScanHuman_8.0 miRDB | 2 | hsa-miR-26b-5p hsa-miR-26a-5p |
| DIANA-MicroT-CDS DIANA-TarBase v9.0 StarBase  TargetScanHuman_8.0 | 2 | hsa-miR-19a-3p hsa-miR-340-5p |
| DIANA-MicroT-CDS TargetScanHuman_8.0 miRDB  miRWalk | 16 | hsa-miR-5002-5p hsa-miR-5692c hsa-miR-4511 hsa-miR-5692b hsa-miR-3927-3p hsa-miR-7856-5p hsa-miR-4330 hsa-miR-6814-5p hsa-miR-4789-3p hsa-miR-4528 hsa-miR-6857-5p hsa-miR-664a-3p hsa-miR-4773 hsa-miR-8063 hsa-miR-6873-3p hsa-miR-4671-3p |
| DIANA-MicroT-CDS StarBase  TargetScanHuman_8.0  miRDB | 6 | hsa-miR-1277-5p hsa-miR-885-5p hsa-miR-23c hsa-miR-186-5p hsa-miR-3163 hsa-miR-3121-3p |
| DIANA-MicroT-CDS  StarBase TargetScanHuman_8.0  miRWalk | 2 | hsa-miR-892c-5p hsa-miR-130a-5p |
| DIANA-TarBase v9.0 StarBase  TargetScanHuman_8.0 miRDB | 1 | hsa-miR-301b-3p |
| StarBase TargetScanHuman_8.0 miRDB  miRWalk | 1 | hsa-miR-4295 |
| DIANA-MicroT-CDS TargetScanHuman_8.0 miRDB | 42 | hsa-miR-5692a hsa-miR-4465 hsa-miR-6868-3p hsa-miR-4282 hsa-miR-194-3p hsa-miR-628-3p hsa-miR-3658 hsa-miR-590-3p hsa-miR-4698 hsa-miR-5696 hsa-miR-190a-3p hsa-miR-6506-5p hsa-miR-4307 hsa-miR-6757-5p hsa-miR-6072 hsa-miR-5704 hsa-miR-135a-5p hsa-miR-4803 hsa-miR-216a-5p hsa-miR-4768-5p hsa-miR-6831-5p hsa-miR-3976 hsa-miR-5683 hsa-miR-664b-3p hsa-miR-4760-3p hsa-miR-7161-3p hsa-miR-548p hsa-miR-5011-5p hsa-miR-3148 hsa-miR-6083 hsa-miR-1297 hsa-miR-1253 hsa-miR-6833-3p hsa-miR-135b-5p hsa-miR-466 hsa-miR-548c-3p hsa-miR-4639-5p hsa-miR-4729 hsa-miR-6080 hsa-miR-579-3p hsa-miR-4775 hsa-miR-6885-3p |
| DIANA-MicroT-CDS TargetScanHuman_8.0 miRWalk | 19 | hsa-miR-6832-5p hsa-miR-1248 hsa-miR-593-3p hsa-miR-3606-3p hsa-miR-6730-5p hsa-miR-1237-3p hsa-miR-548n hsa-miR-4753-3p hsa-miR-4699-5p hsa-miR-6770-5p hsa-miR-4422 hsa-miR-617 hsa-miR-221-3p hsa-miR-585-5p hsa-miR-4716-5p hsa-miR-452-3p hsa-miR-4287 hsa-miR-3136-5p hsa-miR-5580-3p |
| DIANA-MicroT-CDS StarBase  TargetScanHuman_8.0 | 8 | hsa-miR-224-5p hsa-miR-410-3p hsa-miR-5688 hsa-miR-455-5p hsa-miR-5581-3p hsa-miR-577 hsa-miR-300 hsa-miR-495-3p |
| DIANA-TarBase v9.0 StarBase  miRWalk | 5 | hsa-miR-20b-5p hsa-miR-106b-5p hsa-miR-20a-5p hsa-miR-33a-5p hsa-miR-33b-5p |
| DIANA-TarBase v9.0 TargetScanHuman_8.0 miRWalk | 2 | hsa-miR-19b-1-5p hsa-miR-129-2-3p |
| DIANA-TarBase v9.0  StarBase TargetScanHuman_8.0 | 1 | hsa-miR-494-3p |
| TargetScanHuman_8.0 miRDB  miRWalk | 9 | hsa-miR-4314 hsa-miR-4763-5p hsa-miR-7158-3p hsa-miR-589-3p hsa-miR-6891-3p hsa-miR-4796-3p hsa-miR-619-5p hsa-miR-203b-3p hsa-miR-6729-3p |
| StarBase TargetScanHuman_8.0 miRWalk | 4 | hsa-miR-448 hsa-miR-769-5p hsa-miR-668-3p hsa-miR-129-5p |
| DIANA-MicroT-CDS DIANA-TarBase v9.0 | 1 | hsa-miR-148a-5p |
| DIANA-MicroT-CDS miRWalk | 3 | hsa-miR-1298-5p hsa-miR-4774-3p hsa-miR-7152-5p |
| DIANA-MicroT-CDS  TargetScanHuman_8.0 | 39 | hsa-miR-5197-3p hsa-miR-450a-2-3p hsa-miR-2110 hsa-miR-4659a-3p hsa-miR-34a-3p hsa-miR-6818-3p hsa-miR-513a-3p hsa-miR-10a-3p hsa-miR-6815-3p hsa-miR-3692-3p hsa-miR-548az-5p hsa-miR-670-3p hsa-miR-5697 hsa-miR-4762-5p hsa-miR-548e-5p hsa-miR-2052 hsa-miR-1229-5p hsa-miR-548t-5p hsa-miR-1250-3p hsa-miR-4715-5p hsa-miR-153-5p hsa-miR-548x-3p hsa-miR-548aj-3p hsa-miR-222-5p hsa-miR-27a-5p hsa-miR-28-3p hsa-miR-1238-3p hsa-miR-323a-3p hsa-miR-4659b-3p hsa-miR-1305 hsa-miR-936 hsa-miR-452-5p hsa-miR-3156-5p hsa-miR-3667-3p hsa-miR-1468-3p hsa-miR-4662a-3p hsa-miR-548l hsa-miR-513c-3p hsa-miR-3671 |
| DIANA-TarBase v9.0 miRWalk | 26 | hsa-miR-30d-5p hsa-miR-532-3p hsa-miR-32-5p hsa-let-7b-5p hsa-miR-17-3p hsa-let-7g-5p hsa-let-7i-5p hsa-miR-378a-3p hsa-miR-15b-5p hsa-miR-103a-3p hsa-miR-29c-3p hsa-let-7d-5p hsa-miR-34c-5p hsa-miR-185-5p hsa-miR-873-5p hsa-miR-378c hsa-miR-324-3p hsa-miR-125b-5p hsa-miR-25-5p hsa-let-7e-5p hsa-let-7a-5p hsa-let-7c-5p hsa-miR-203a-3p hsa-miR-204-5p hsa-miR-183-5p hsa-miR-191-5p |
| DIANA-TarBase v9.0 StarBase | 3 | hsa-miR-17-5p hsa-miR-106a-5p hsa-miR-93-5p |
| DIANA-TarBase v9.0 TargetScanHuman_8.0 | 16 | hsa-miR-34b-5p hsa-miR-627-5p hsa-miR-142-5p hsa-miR-532-5p hsa-miR-31-5p hsa-miR-4745-5p hsa-miR-132-3p hsa-miR-335-3p hsa-miR-196a-5p hsa-miR-196b-5p hsa-miR-30e-3p hsa-miR-212-3p hsa-miR-802 hsa-miR-181a-5p hsa-miR-21-5p hsa-miR-27b-3p |
| miRDB  miRWalk | 2 | hsa-miR-103a-1-5p hsa-miR-12116 |
| TargetScanHuman_8.0 miRDB | 32 | hsa-miR-374b-3p hsa-miR-103a-2-5p hsa-miR-3977 hsa-miR-4328 hsa-miR-302a-5p hsa-miR-6880-5p hsa-miR-188-3p hsa-miR-708-3p hsa-miR-4789-5p hsa-miR-548f-5p hsa-miR-944 hsa-miR-369-3p hsa-miR-4694-3p hsa-miR-5007-5p hsa-miR-4477b hsa-miR-6077 hsa-miR-548x-5p hsa-miR-548aj-5p hsa-miR-6894-3p hsa-miR-4666b hsa-miR-4796-5p hsa-miR-6875-3p hsa-miR-1261 hsa-miR-607 hsa-miR-548b-3p hsa-miR-7976 hsa-miR-4724-5p hsa-miR-659-3p hsa-miR-33a-3p hsa-miR-548g-5p hsa-miR-4780 hsa-miR-5582-5p |
| StarBase  miRWalk | 3 | hsa-miR-1276 hsa-miR-4524a-5p hsa-miR-490-3p |
| TargetScanHuman_8.0 miRWalk | 109 | hsa-miR-520f-3p hsa-miR-1298-3p hsa-miR-5682 hsa-miR-6740-5p hsa-miR-6793-3p hsa-miR-500a-5p hsa-let-7b-3p hsa-miR-551b-5p hsa-miR-8071 hsa-miR-623 hsa-miR-875-3p hsa-miR-129-1-3p hsa-miR-7150 hsa-miR-6504-3p hsa-miR-6810-5p hsa-miR-4685-3p hsa-miR-4652-3p hsa-miR-4804-3p hsa-miR-3678-3p hsa-miR-1260a hsa-miR-125b-2-3p hsa-miR-1236-3p hsa-miR-4720-3p hsa-miR-197-5p hsa-miR-6128 hsa-miR-6859-5p hsa-miR-1291 hsa-miR-3162-5p hsa-miR-6854-5p hsa-miR-1323 hsa-miR-4292 hsa-miR-6795-3p hsa-miR-4299 hsa-miR-512-3p hsa-miR-331-5p hsa-miR-198 hsa-miR-612 hsa-miR-935 hsa-miR-6883-3p hsa-miR-6775-3p hsa-miR-6782-3p hsa-miR-6867-3p hsa-miR-6858-3p hsa-miR-6512-3p hsa-miR-1914-5p hsa-miR-3945 hsa-miR-374a-3p hsa-miR-4450 hsa-miR-6862-5p hsa-miR-3064-3p hsa-miR-4251 hsa-miR-6734-3p hsa-miR-6835-3p hsa-miR-3691-3p hsa-miR-556-3p hsa-miR-520d-5p hsa-miR-6884-3p hsa-miR-8081 hsa-miR-5586-3p hsa-miR-500b-5p hsa-miR-1266-3p hsa-miR-589-5p hsa-miR-624-3p hsa-miR-650 hsa-miR-216a-3p hsa-miR-7-1-3p hsa-miR-6860 hsa-miR-890 hsa-miR-3154 hsa-miR-493-5p hsa-miR-4423-5p hsa-miR-6780b-3p hsa-miR-4443 hsa-miR-548an hsa-miR-7157-3p hsa-miR-6886-3p hsa-miR-524-5p hsa-miR-6806-5p hsa-miR-454-5p hsa-miR-570-3p hsa-miR-7113-3p hsa-miR-204-3p hsa-miR-4676-5p hsa-miR-526b-5p hsa-miR-222-3p hsa-miR-1260b hsa-miR-3198 hsa-miR-4646-5p hsa-miR-4712-3p hsa-miR-3187-5p hsa-miR-450b-5p hsa-miR-3654 hsa-miR-6868-5p hsa-miR-19b-2-5p hsa-miR-583 hsa-miR-1227-3p hsa-miR-6737-3p hsa-miR-32-3p hsa-miR-6747-3p hsa-miR-1827 hsa-miR-374c-3p hsa-miR-3129-3p hsa-miR-6507-5p hsa-miR-7978 hsa-miR-5008-3p hsa-miR-205-3p hsa-miR-5693 hsa-miR-548as-3p hsa-miR-433-3p |
| StarBase TargetScanHuman_8.0 | 7 | hsa-miR-342-3p hsa-miR-376a-3p hsa-miR-384 hsa-miR-3129-5p hsa-miR-376b-3p hsa-miR-199a-3p hsa-miR-199b-3p |
| DIANA-MicroT-CDS | 10 | hsa-miR-627-3p hsa-miR-4778-3p hsa-miR-5094 hsa-miR-3152-5p hsa-miR-4275 hsa-miR-7702 hsa-miR-9-5p hsa-miR-5681a hsa-miR-498 hsa-miR-4766-5p |
| DIANA-TarBase v9.0 | 59 | hsa-miR-576-3p hsa-miR-92b-3p hsa-miR-205-5p hsa-miR-223-3p hsa-miR-449a hsa-miR-1-3p hsa-miR-200a-5p hsa-miR-1301-3p hsa-miR-584-5p hsa-miR-30a-5p hsa-miR-582-5p hsa-miR-92a-1-5p hsa-miR-148b-3p hsa-miR-141-5p hsa-miR-375-3p hsa-miR-4521 hsa-miR-148b-5p hsa-miR-30b-5p kshv-miR-K12-9-3p hsa-miR-139-5p kshv-miR-K12-10b hsa-miR-126-3p hsa-let-7f-5p hsa-miR-30e-5p hsa-miR-25-3p hsa-miR-423-5p hsa-miR-219a-1-3p hsa-miR-625-5p hsa-miR-155-5p ebv-miR-BART2-5p hsa-miR-16-1-3p hsa-miR-181a-2-3p hsa-miR-18a-5p hsa-miR-101-3p hsa-miR-3934-5p kshv-miR-K12-10a-3p hsa-miR-30c-5p hsa-miR-193b-5p hsa-miR-29b-3p ebv-miR-BART15 hsa-miR-92a-3p hsa-miR-660-5p hsa-miR-151a-3p hsa-miR-148a-3p hsa-miR-98-5p hsa-miR-29a-3p hsa-miR-15a-5p hsa-miR-147a hsa-miR-449b-5p hsa-miR-195-5p hsa-miR-424-5p hsa-miR-194-5p hsa-miR-34a-5p hsa-miR-99b-3p hsa-miR-192-5p hsa-miR-107 hsa-miR-188-5p hsa-miR-16-5p hsa-miR-210-3p |
| miRDB | 6 | hsa-miR-10397-3p hsa-miR-12133 hsa-miR-6529-5p hsa-miR-520e-5p hsa-miR-12117 hsa-miR-12136 |
| miRWalk | 593 | hsa-miR-522-5p hsa-miR-5088-5p hsa-miR-665 hsa-miR-518d-5p hsa-miR-4740-5p hsa-miR-4493 hsa-miR-6887-5p hsa-miR-4709-3p hsa-miR-4524b-3p hsa-miR-4730 hsa-miR-302b-3p hsa-miR-526a-3p hsa-miR-3155a hsa-miR-648 hsa-miR-377-5p hsa-miR-5196-3p hsa-miR-2114-3p hsa-miR-6499-3p hsa-miR-2276-3p hsa-miR-489-3p hsa-miR-370-5p hsa-miR-675-3p hsa-miR-6760-3p hsa-miR-7162-5p hsa-miR-483-3p hsa-miR-4486 hsa-miR-4534 hsa-miR-6834-3p hsa-miR-6759-3p hsa-miR-4747-5p hsa-miR-6813-3p hsa-miR-26a-2-3p hsa-miR-3122 hsa-miR-758-3p hsa-miR-6858-5p hsa-miR-378d hsa-miR-6765-5p hsa-miR-6511a-5p hsa-miR-6748-3p hsa-miR-4463 hsa-miR-6132 hsa-miR-518e-5p hsa-miR-3917 hsa-miR-320c hsa-miR-6836-5p hsa-miR-6503-5p hsa-miR-3162-3p hsa-miR-6797-5p hsa-miR-6504-5p hsa-miR-4722-5p hsa-miR-424-3p hsa-miR-5186 hsa-miR-3618 hsa-miR-1238-5p hsa-miR-5196-5p hsa-miR-6803-3p hsa-miR-664b-5p hsa-miR-6511a-3p hsa-miR-486-3p hsa-miR-507 hsa-miR-4765 hsa-miR-7113-5p hsa-miR-766-3p hsa-miR-3908 hsa-miR-4761-5p hsa-miR-186-3p hsa-miR-489-5p hsa-miR-92b-5p hsa-miR-6769b-3p hsa-miR-7106-3p hsa-miR-6829-3p hsa-miR-6766-3p hsa-miR-5584-5p hsa-miR-943 hsa-miR-6883-5p hsa-miR-6890-3p hsa-miR-3149 hsa-miR-4715-3p hsa-miR-4731-5p hsa-miR-4750-5p hsa-miR-191-3p hsa-miR-6841-3p hsa-miR-3186-3p hsa-miR-6845-5p hsa-miR-544b hsa-miR-5706 hsa-miR-4633-3p hsa-miR-6778-5p hsa-miR-3938 hsa-miR-7848-3p hsa-miR-371b-3p hsa-miR-4491 hsa-miR-4751 hsa-miR-3074-3p hsa-miR-6716-3p hsa-miR-298 hsa-miR-4326 hsa-miR-6856-3p hsa-miR-10396a-3p hsa-miR-6807-5p hsa-miR-199a-5p hsa-miR-6735-5p hsa-miR-3682-3p hsa-miR-6828-5p hsa-miR-4689 hsa-miR-519a-3p hsa-miR-9718 hsa-miR-4739 hsa-miR-630 hsa-miR-600 hsa-miR-371a-3p hsa-miR-4436b-3p hsa-miR-3150a-5p hsa-miR-338-5p hsa-miR-766-5p hsa-miR-211-3p hsa-miR-887-3p hsa-miR-3689a-5p hsa-miR-1228-3p hsa-miR-491-5p hsa-miR-548al hsa-miR-10523-5p hsa-miR-320e hsa-miR-641 hsa-miR-4433a-5p hsa-miR-4312 hsa-miR-4316 hsa-miR-5006-5p hsa-miR-4690-5p hsa-miR-5699-5p hsa-miR-4706 hsa-miR-4297 hsa-miR-3680-3p hsa-miR-2392 hsa-miR-5690 hsa-miR-4418 hsa-miR-378f hsa-miR-6772-5p hsa-miR-223-5p hsa-miR-4640-3p hsa-miR-4680-5p hsa-miR-504-3p hsa-miR-149-3p hsa-miR-1255b-5p hsa-miR-5708 hsa-miR-6812-3p hsa-miR-4731-3p hsa-miR-765 hsa-miR-1911-5p hsa-miR-4654 hsa-miR-21-3p hsa-miR-550b-2-5p hsa-miR-6782-5p hsa-miR-2355-3p hsa-miR-2117 hsa-miR-6864-3p hsa-miR-100-5p hsa-miR-6774-5p hsa-miR-6801-5p hsa-miR-4263 hsa-miR-6808-3p hsa-miR-6500-3p hsa-miR-511-5p hsa-miR-4673 hsa-miR-526a-5p hsa-miR-3199 hsa-miR-152-3p hsa-miR-4759 hsa-miR-3169 hsa-miR-6757-3p hsa-miR-6821-3p hsa-miR-4728-5p hsa-miR-4539 hsa-miR-520c-3p hsa-miR-518a-5p hsa-miR-608 hsa-miR-6514-5p hsa-miR-770-5p hsa-miR-509-3p hsa-miR-4496 hsa-miR-4782-5p hsa-miR-449b-3p hsa-miR-500b-3p hsa-miR-1226-3p hsa-miR-6779-3p hsa-miR-6808-5p hsa-miR-200b-5p hsa-miR-3157-3p hsa-miR-324-5p hsa-miR-5195-5p hsa-miR-3713 hsa-miR-26a-1-3p hsa-miR-4520-5p hsa-miR-122-3p hsa-miR-6874-5p hsa-miR-4750-3p hsa-miR-10b-5p hsa-miR-7705 hsa-miR-1843 hsa-miR-1343-5p hsa-miR-610 hsa-miR-541-3p hsa-miR-8089 hsa-miR-548aq-5p hsa-miR-3130-5p hsa-miR-1258 hsa-miR-488-5p hsa-miR-6881-3p hsa-miR-3142 hsa-miR-6508-3p hsa-miR-5193 hsa-miR-7843-5p hsa-miR-6781-3p hsa-miR-4259 hsa-miR-491-3p hsa-miR-6882-3p hsa-miR-12114 hsa-miR-4724-3p hsa-miR-7160-3p hsa-miR-6744-5p hsa-miR-873-3p hsa-miR-3684 hsa-miR-1257 hsa-miR-670-5p hsa-miR-4429 hsa-miR-4667-5p hsa-miR-4728-3p hsa-miR-4524b-5p hsa-miR-6797-3p hsa-miR-330-5p hsa-miR-10401-3p hsa-miR-6829-5p hsa-miR-340-3p hsa-miR-3158-5p hsa-miR-451a hsa-miR-3194-3p hsa-miR-7152-3p hsa-miR-4476 hsa-miR-4498 hsa-miR-4472 hsa-let-7i-3p hsa-miR-501-5p hsa-miR-5702 hsa-miR-199b-5p hsa-miR-383-3p hsa-miR-6758-5p hsa-miR-6879-3p hsa-miR-527 hsa-miR-4270 hsa-miR-450a-1-3p hsa-miR-519a-5p hsa-miR-2116-5p hsa-miR-375-5p hsa-miR-329-3p hsa-miR-6511b-5p hsa-miR-3675-3p hsa-miR-4442 hsa-miR-4514 hsa-miR-6856-5p hsa-miR-1255b-2-3p hsa-miR-378b hsa-miR-486-5p hsa-miR-3936 hsa-miR-4782-3p hsa-miR-6823-3p hsa-miR-6820-3p hsa-miR-4656 hsa-miR-4321 hsa-miR-1587 hsa-miR-4725-5p hsa-miR-1236-5p hsa-miR-764 hsa-miR-6866-3p hsa-miR-518f-5p hsa-miR-548au-3p hsa-miR-9985 hsa-miR-6880-3p hsa-miR-379-3p hsa-miR-122-5p hsa-miR-4530 hsa-miR-24-1-5p hsa-miR-3689e hsa-miR-12131 hsa-miR-5584-3p hsa-miR-652-5p hsa-miR-485-3p hsa-miR-4684-3p hsa-miR-6728-5p hsa-miR-4437 hsa-miR-455-3p hsa-miR-211-5p hsa-miR-4313 hsa-miR-3184-3p hsa-miR-101-2-5p hsa-miR-3679-3p hsa-miR-137-5p hsa-miR-7850-5p hsa-miR-6882-5p hsa-miR-514a-5p hsa-miR-3190-5p hsa-miR-639 hsa-miR-1908-3p hsa-miR-6811-3p hsa-miR-1183 hsa-miR-520g-5p hsa-miR-7854-3p hsa-miR-5004-5p hsa-miR-1275 hsa-miR-500a-3p hsa-miR-1288-5p hsa-miR-9898 hsa-miR-4646-3p hsa-miR-3529-5p hsa-miR-5090 hsa-miR-1-5p hsa-miR-7154-3p hsa-miR-6792-5p hsa-miR-671-5p hsa-miR-3124-3p hsa-miR-7108-3p hsa-miR-502-3p hsa-miR-3170 hsa-miR-4754 hsa-miR-4306 hsa-miR-3689b-5p hsa-miR-3918 hsa-miR-373-3p hsa-miR-6503-3p hsa-miR-5698 hsa-miR-2114-5p hsa-miR-922 hsa-miR-6766-5p hsa-miR-4732-3p hsa-miR-6892-3p hsa-miR-4797-5p hsa-miR-6824-5p hsa-miR-125a-5p hsa-miR-6839-5p hsa-miR-378e hsa-miR-4800-3p hsa-miR-6513-3p hsa-miR-493-3p hsa-miR-6809-5p hsa-miR-1247-5p hsa-miR-510-5p hsa-miR-5587-3p hsa-miR-4669 hsa-miR-6889-5p hsa-miR-193b-3p hsa-miR-3620-3p hsa-miR-4265 hsa-miR-6861-3p hsa-miR-520a-3p hsa-miR-320a-5p hsa-miR-6836-3p hsa-miR-4520-3p hsa-miR-6793-5p hsa-miR-24-3p hsa-miR-6825-5p hsa-miR-134-5p hsa-miR-3190-3p hsa-miR-6070 hsa-miR-4430 hsa-miR-3065-3p hsa-miR-365a-5p hsa-miR-380-3p hsa-miR-6805-5p hsa-let-7e-3p hsa-miR-659-5p hsa-miR-5588-5p hsa-miR-3180-3p hsa-miR-6529-3p hsa-miR-1288-3p hsa-miR-3679-5p hsa-miR-411-3p hsa-miR-4494 hsa-miR-6867-5p hsa-miR-128-1-5p hsa-miR-4645-3p hsa-miR-1266-5p hsa-miR-409-5p hsa-miR-5787 hsa-miR-147b-3p hsa-miR-4761-3p hsa-miR-5585-3p hsa-miR-3683 hsa-miR-10392-5p hsa-miR-6804-3p hsa-miR-1304-5p hsa-miR-550a-5p hsa-miR-3605-5p hsa-miR-6129 hsa-miR-299-5p hsa-miR-215-3p hsa-miR-5691 hsa-miR-516a-5p hsa-miR-4713-3p hsa-miR-8052 hsa-miR-4451 hsa-miR-6768-5p hsa-miR-1249-5p hsa-miR-6852-5p hsa-miR-6510-3p hsa-miR-520a-5p hsa-miR-3180 hsa-miR-378g hsa-miR-3155b hsa-miR-518c-5p hsa-miR-646 hsa-miR-26b-3p hsa-miR-523-5p hsa-miR-6715b-5p hsa-miR-1281 hsa-miR-5192 hsa-miR-4433a-3p hsa-miR-6851-3p hsa-miR-622 hsa-miR-7156-3p hsa-miR-5001-3p hsa-miR-6748-5p hsa-miR-362-5p hsa-miR-549a-5p hsa-miR-7156-5p hsa-miR-3675-5p hsa-miR-619-3p hsa-miR-4701-5p hsa-miR-4752 hsa-miR-5093 hsa-miR-96-3p hsa-miR-4441 hsa-miR-4535 hsa-miR-3184-5p hsa-miR-4633-5p hsa-miR-4290 hsa-miR-345-3p hsa-miR-502-5p hsa-miR-5187-3p hsa-miR-591 hsa-miR-4753-5p hsa-miR-6875-5p hsa-miR-134-3p hsa-miR-6769b-5p hsa-miR-6847-5p hsa-miR-4655-3p hsa-miR-1538 hsa-miR-30c-2-3p hsa-miR-3116 hsa-miR-5190 hsa-miR-520f-5p hsa-miR-20b-3p hsa-miR-4636 hsa-miR-4652-5p hsa-miR-4318 hsa-miR-4490 hsa-miR-4260 hsa-miR-1262 hsa-miR-30c-1-3p hsa-miR-548w hsa-miR-4786-3p hsa-miR-23b-5p hsa-miR-596 hsa-miR-371b-5p hsa-miR-940 hsa-miR-519d-5p hsa-miR-4653-5p hsa-miR-302c-3p hsa-miR-184 hsa-miR-93-3p hsa-miR-5087 hsa-miR-6800-3p hsa-miR-11181-3p hsa-miR-6814-3p hsa-miR-450b-3p hsa-miR-6798-5p hsa-let-7a-2-3p hsa-miR-6820-5p hsa-miR-4447 hsa-miR-6752-5p hsa-miR-4271 hsa-miR-6747-5p hsa-miR-6737-5p hsa-miR-7114-3p hsa-miR-12125 hsa-miR-636 hsa-miR-7851-3p hsa-miR-520c-5p hsa-miR-6881-5p hsa-miR-934 hsa-miR-3178 hsa-miR-4651 hsa-miR-5189-3p hsa-miR-4781-3p hsa-miR-4518 hsa-miR-609 hsa-miR-3609 hsa-miR-4440 hsa-miR-3664-3p hsa-miR-551a hsa-miR-891a-3p hsa-miR-6838-3p hsa-miR-6127 hsa-miR-6806-3p hsa-miR-3144-3p hsa-miR-4720-5p hsa-miR-4510 hsa-miR-8088 hsa-miR-760 hsa-let-7c-3p hsa-miR-4298 hsa-miR-611 hsa-miR-92a-2-5p hsa-miR-1256 hsa-miR-4755-3p hsa-miR-1224-3p hsa-miR-125b-1-3p hsa-miR-4774-5p hsa-miR-1307-5p hsa-miR-4435 hsa-miR-4500 hsa-miR-6798-3p hsa-miR-4266 hsa-miR-1910-5p hsa-miR-711 hsa-miR-6769a-5p hsa-miR-7843-3p hsa-miR-4701-3p hsa-miR-7162-3p hsa-miR-6131 hsa-miR-6731-5p hsa-miR-6845-3p hsa-miR-320b hsa-miR-122b-5p hsa-miR-297 hsa-miR-6781-5p hsa-miR-9902 hsa-miR-1283 hsa-miR-629-3p hsa-miR-5006-3p hsa-miR-4319 hsa-miR-331-3p hsa-miR-5699-3p hsa-miR-6746-3p hsa-miR-4433b-3p hsa-miR-523-3p hsa-miR-4770 hsa-miR-6849-5p hsa-miR-8083 hsa-miR-3115 hsa-miR-4804-5p hsa-miR-4733-3p hsa-miR-4507 hsa-miR-4658 hsa-miR-7845-5p hsa-miR-4516 hsa-miR-4475 hsa-miR-518c-3p hsa-miR-1910-3p hsa-miR-4681 hsa-miR-1249-3p hsa-miR-6742-3p hsa-miR-519b-5p hsa-miR-6879-5p hsa-miR-6796-3p hsa-miR-6790-5p hsa-miR-7973 hsa-miR-501-3p hsa-miR-574-5p hsa-miR-1909-5p hsa-miR-103b hsa-miR-4657 hsa-miR-519c-5p hsa-miR-3141 hsa-miR-197-3p hsa-miR-12113 hsa-miR-8085 hsa-miR-24-2-5p hsa-miR-5582-3p hsa-miR-145-5p hsa-miR-4800-5p hsa-miR-6086 hsa-miR-6846-3p hsa-miR-6817-5p hsa-miR-668-5p hsa-miR-5591-3p hsa-miR-10401-5p hsa-miR-613 hsa-miR-1293 hsa-miR-4785 hsa-miR-6830-5p hsa-miR-7706 hsa-miR-1225-3p hsa-miR-4294 hsa-miR-302d-3p |
| StarBase | 12 | hsa-miR-487a-3p hsa-miR-3200-5p hsa-miR-543 hsa-miR-190b hsa-miR-153-3p hsa-miR-376c-3p hsa-miR-218-5p hsa-miR-514b-5p hsa-miR-519d-3p hsa-miR-516b-5p hsa-miR-190a-5p hsa-miR-513c-5p |
| TargetScanHuman_8.0 | 296 | hsa-miR-2682-5p hsa-miR-4743-3p hsa-miR-381-3p hsa-miR-6895-3p hsa-miR-3682-5p hsa-miR-6515-3p hsa-miR-3681-3p hsa-miR-146a-5p hsa-let-7a-3p hsa-miR-3653-3p hsa-miR-4274 hsa-miR-548ar-3p hsa-miR-4668-5p hsa-miR-6771-3p hsa-miR-4513 hsa-miR-575 hsa-miR-653-5p hsa-miR-4477a hsa-miR-95-5p hsa-miR-3173-5p hsa-miR-5583-5p hsa-miR-330-3p hsa-miR-124-3p.1 hsa-miR-3910 hsa-miR-146b-3p hsa-miR-20a-3p hsa-miR-370-3p hsa-miR-329-5p hsa-miR-8055 hsa-miR-3612 hsa-miR-3192-3p hsa-miR-1231 hsa-miR-4255 hsa-miR-5680 hsa-miR-1207-5p hsa-miR-302b-5p hsa-miR-939-3p hsa-miR-136-3p hsa-miR-576-5p hsa-miR-586 hsa-miR-4693-3p hsa-miR-4419b hsa-miR-496.2 hsa-miR-302c-3p.2 hsa-miR-6853-5p hsa-miR-511-3p hsa-miR-6887-3p hsa-miR-1285-3p hsa-miR-548ba hsa-miR-3153 hsa-miR-30a-3p hsa-miR-548d-3p hsa-miR-8080 hsa-miR-4717-5p hsa-miR-605-3p hsa-miR-3161 hsa-miR-3591-5p hsa-miR-548h-3p hsa-miR-548t-3p hsa-miR-4489 hsa-miR-5011-3p hsa-miR-3119 hsa-miR-4740-3p hsa-miR-6715a-3p hsa-miR-4276 hsa-miR-580-5p hsa-miR-6715b-3p hsa-miR-599 hsa-miR-216b-3p hsa-miR-4682 hsa-miR-6837-3p hsa-miR-4519 hsa-miR-4666a-5p hsa-miR-885-3p hsa-miR-495-5p hsa-miR-4676-3p hsa-miR-548ae-3p hsa-miR-124-3p.2 hsa-miR-6857-3p hsa-miR-216b-5p hsa-miR-4253 hsa-miR-6817-3p hsa-miR-5004-3p hsa-miR-654-3p hsa-miR-5096 hsa-miR-98-3p hsa-miR-325 hsa-miR-4280 hsa-miR-548aa hsa-miR-7110-3p hsa-miR-3132 hsa-miR-4503 hsa-miR-4999-3p hsa-miR-126-5p hsa-miR-497-3p hsa-miR-548bb-3p hsa-miR-4662b hsa-miR-6826-3p hsa-miR-4524a-3p hsa-miR-9-3p hsa-miR-3646 hsa-miR-146b-5p hsa-miR-548ah-3p hsa-miR-548q hsa-miR-4478 hsa-miR-128-3p hsa-miR-548ap-3p hsa-miR-6855-3p hsa-miR-548ag hsa-miR-7151-5p hsa-miR-3125 hsa-miR-1229-3p hsa-miR-1284 hsa-miR-3185 hsa-miR-4705 hsa-miR-759 hsa-miR-548ax hsa-miR-144-3p hsa-miR-520h hsa-miR-141-3p hsa-miR-3117-5p hsa-miR-1279 hsa-miR-142-3p.2 hsa-miR-4501 hsa-miR-130b-5p hsa-miR-6799-3p hsa-miR-4272 hsa-miR-632 hsa-miR-3942-3p hsa-miR-4289 hsa-miR-3688-3p hsa-miR-548aq-3p hsa-miR-5191 hsa-miR-181c-5p hsa-miR-499b-5p hsa-miR-200a-3p hsa-miR-548e-3p hsa-miR-548ac hsa-miR-5694 hsa-miR-6514-3p hsa-miR-4736 hsa-miR-5088-3p hsa-miR-570-5p hsa-miR-891b hsa-miR-1252-3p hsa-miR-6809-3p hsa-miR-27a-3p hsa-miR-1285-5p hsa-miR-539-5p hsa-miR-8084 hsa-miR-3916 hsa-miR-522-3p hsa-miR-889-5p hsa-miR-7-2-3p hsa-miR-3123 hsa-miR-4283 hsa-miR-548o-3p hsa-miR-548f-3p hsa-miR-892c-3p hsa-miR-6507-3p hsa-miR-4456 hsa-miR-5009-3p hsa-miR-6763-5p hsa-miR-6516-5p hsa-miR-4267 hsa-miR-647 hsa-miR-4426 hsa-miR-7853-5p hsa-miR-3192-5p hsa-miR-6869-5p hsa-miR-383-5p.2 hsa-miR-6888-3p hsa-miR-5590-3p hsa-miR-655-3p hsa-miR-181d-5p hsa-miR-548m hsa-miR-6733-5p hsa-miR-8068 hsa-miR-6505-5p hsa-miR-6816-3p hsa-miR-1295b-3p hsa-miR-7159-3p hsa-miR-4439 hsa-miR-514a-3p hsa-miR-3925-5p hsa-miR-4697-3p hsa-miR-181b-5p hsa-miR-6509-5p hsa-miR-6791-5p hsa-miR-6893-3p hsa-miR-1303 hsa-miR-4417 hsa-miR-5589-3p hsa-miR-183-5p.2 hsa-miR-4469 hsa-miR-510-3p hsa-miR-4747-3p hsa-miR-3151-3p hsa-miR-6074 hsa-miR-142-3p.1 hsa-miR-1295a hsa-miR-6800-5p hsa-miR-4687-3p hsa-miR-3685 hsa-miR-10b-3p hsa-miR-5579-3p hsa-miR-5100 hsa-miR-6516-3p hsa-miR-4763-3p hsa-miR-29a-5p hsa-miR-7153-5p hsa-miR-590-5p hsa-miR-6794-3p hsa-miR-4262 hsa-miR-5571-3p hsa-miR-3177-5p hsa-miR-520g-3p hsa-miR-3126-3p hsa-miR-1273h-3p hsa-miR-2115-3p hsa-miR-382-3p hsa-miR-302d-5p hsa-miR-4309 hsa-miR-19a-5p hsa-miR-185-3p hsa-miR-1243 hsa-miR-4481 hsa-miR-7153-3p hsa-miR-633 hsa-miR-4795-3p hsa-miR-4427 hsa-miR-3714 hsa-miR-3651 hsa-miR-545-5p hsa-miR-449c-5p hsa-miR-3913-3p hsa-miR-548j-3p hsa-miR-651-3p hsa-miR-7847-3p hsa-miR-224-3p hsa-miR-3152-3p hsa-miR-580-3p hsa-miR-6739-5p hsa-miR-1206 hsa-miR-548am-3p hsa-miR-6842-3p hsa-miR-548a-3p hsa-miR-3973 hsa-miR-7844-5p hsa-miR-4670-3p hsa-miR-5189-5p hsa-let-7f-1-3p hsa-miR-100-3p hsa-miR-3929 hsa-miR-105-5p hsa-miR-30d-3p hsa-miR-4288 hsa-miR-1245b-3p hsa-miR-6728-3p hsa-miR-4647 hsa-miR-3165 hsa-miR-1299 hsa-miR-7-5p hsa-miR-412-3p hsa-miR-548z hsa-miR-377-3p hsa-miR-374c-5p hsa-miR-874-3p hsa-miR-4639-3p hsa-miR-3150a-3p hsa-miR-924 hsa-miR-4473 hsa-miR-218-1-3p hsa-miR-5003-5p hsa-miR-3156-3p hsa-miR-409-3p hsa-miR-4666a-3p hsa-miR-548az-3p hsa-miR-2054 hsa-miR-889-3p hsa-miR-15a-3p hsa-miR-548ai hsa-miR-3614-3p hsa-miR-548ao-5p hsa-miR-4461 hsa-miR-6720-5p hsa-miR-649 hsa-miR-8066 hsa-miR-3935 hsa-miR-3529-3p hsa-miR-2355-5p hsa-miR-6754-3p hsa-miR-6124 hsa-miR-3159 hsa-miR-514b-3p hsa-miR-4643 |
