# Supplementary material for: DEP domain containing 1: a potential oncogenic driver
Source: Biol Res. 2025 Oct 8;58:64. doi: 10.1186/s40659-025-00643-0 (PMC12505635; doi:10.1186/s40659-025-00643-0)
Supplement: Supplementary file 7 — Supplementary Material 7 [file 40659_2025_643_MOESM7_ESM.docx]

**Figure S1. The** **gene model of DEPDC1.** DEPDC1 has 5 transcripts, consists of different exons and different number of exons. The gene model of each transcript is show in the above image. The information and data were obtained from GTEx database (analyzed on 01/19/2025, https://www.gtexportal.org/home/).

**Figure S2. Isoform expression profile of DEPDC1 in different normal human tissues.** The relative expression of nineteen DEPDC1 isoforms is separately presented in different human organ tissues.The data source is GTEx Analysis Release V8 (performed on 01/19/2025, https://www.gtex

portal.org/home/).

**Figure S3. The expression of DEPDC1 in distinct single cell types based on scRNA-seq of human tissues.** The analysis is performed with The Human Protein Atlas database (HPA,https: //www.proteinatlas.org/, analyzed on 01/19/2025), wherein the single cell transcriptomics data were respectively obtained from the Single Cell Expression Atlas (<https://www>.ebi.ac.uk/gxa/sc/home),

the Human Cell Atlas (https://www.humancellatlas.org/), the Gene Expression Omnibus (https://www.ncbi.nlm.nih.gov/geo/), the Allen Brain Map(https://portal.brain-map.org/), and the European Genome-phenome Archive (https://ega-archive.org/).

**Figure S4-1. Expression profiles of DEPDC1 in cancers.** RNA levels of DEPDC1 in normal human tissues (A) and cells (B) based on HPA datasets. (C) Violin plots showing DEPDC1 expression levels in various human normal tissues based on GTEx datasets. (D) DEPDC1 protein expression in normal tissues in the HPA datasets.

**Figure S4-2. Expression profiles of DEPDC1 in cancers.** (A) The differential expression of DEPDC1 mRNA in tumor tissues compared with non-tumor tissues are examined in distinct cancers base on RNA-seq data from TCGA. (B) Paired-sample analysis of DEPDC1 expression in several cancer types based on TCGA datasets. (C) Expression of DEPDC1 in cancer cells. (D) DEPDC1 protein expression in tumor tissues. Significance is indicated as follows: **p*<0.05, ***p*<0.01, and ****p*<0.001.

**Figure S5. Analysis of** **disease-specific survival based on DEPDC1 expression in cancers.** Patients are classified into DEPDC1^high^ (>median) and DEPDC1^low^ (<median) groups for each cancer type listed in TCGA.

**Figure S6**. **Analysis of progression-free interval based on DEPDC1 expression in cancers**. Patients are classified into DEPDC1 ^high^ (>median) and DEPDC1 ^low^ (<median) groups for each cancer type listed in TCGA.

**Figure S7. Prognostic value of DEPDC1 in cancers according to KM-plotter analysis.** KM-plotter analyses have been performed on (A) overall survival (OS), (B) disease-specific survival (DSS) and (C) progression-free interval (PFI) on the basis of DEPDC1 expression.

**Figure S8. Time-dependent ROC curve analysis based on DEPDC1 expression in cancers**. The prognostic value of DEPDC1 is evaluated for several intervals (1 year, 3 year and 5 year) for overall (A), disease-specific (B) and progression-free survival (C). Original data are from TCGA.

**Figure S9. Higher levels of DEPDC1 expression are associated with overall survival,** **disease-free survival (DFS), progression-free survival (PFS), and recurrence-free survival (RFS) in multiple external datasets.**

**Figure S10. Relevance of DEPDC1 across different functional states in distinct cancers**. Through the analysis of the CancerSEA database, the functional roles of DEPDC1 in different cancers has been characterized at the single cell level. The bar chart indicates the number of datasets in which DEPDC1 significantly correlates the specific functional states. Average correlations between DEPDC1 and specific functional states in different cancers are presented as dots (red: positive correlation, blue: negative correlation).

**Figure S11-1. GSEA-based analysis of cell proliferation-related pathways correlating with** **DEPDC1 gene expression in 36 cancer types**. The enrichment results indicate that genes involved in cell proliferation related pathways, such as NOTCH(A), PLK1(B), PI3K/AKT (C), NF-κB (D), TGF-β (E), Hedgehog (F), MYC (G), MAPK (H), or WNT (I) related signaling pathways, are positively enriched in cancesr expressing high DEPDC1 mRNA levels in the majority of cancer types.

**Figure S11-2. GSEA-based analysis of cell proliferation related pathways correlating with DEPDC1 gene expression in 36 cancer types**. The enrichment results indicate that genes involved in cell proliferation related pathways, such as HIPPO (A), P53 (B), P27 (C), MCM (D), or RBE2F (E) related signaling pathways, are positively enriched cancers expressing high DEPDC1 mRNA levels in the majority of cancer types except COAD (F), CHOL (G) and SKCM (H).

**Figure S12. Correlation analysis of DEPDC1 co-expressed genes in pan-cancers.** The correlation of DEPDC1-related co-expressed genes (by Sperman’s correlaiton coefficient, r) are presented as correlation heatmaps in the 36 cancer types. Statistical significance is shown as **p*<0.05, ***p*<0.01, ***p<0.001.

**Figure S13-1. Differential expression of DEPDC1 co-expressed genes in 36 cancer types based on DEPDC1 expression**. The expression levels of DEPDC1 co-expressed genes were presented in ACC (A), BLCA (B), BRCA (C), CESC (D), CHOL (E), COAD (F), DLBC (G), ESAD (H), and ESCA (I) as heatmaps grouped (High: >median Low: <median). **p*<0.05, ***p*<0.01, ***p<0.001.

**Figure S13-2. Differential expression of DEPDC1 co-expressed genes in 36 cancer types.** The expression levels of DEPDC1 co-expressed genes in ESCC (A), GBM (B), HNSC (C), KICH (D), KIRC (E), KIRP (F), LAML (G), LGG (H), and LIHC (I) are presented as co-expression heatmaps grouped (High: >median Low: <median), **p*<0.05, ***p*<0.01, ***p<0.001.

**Figure S13-3. Differential expression of DEPDC1 co-expressed genes in 36 cancer types.** The expression levels of DEPDC1 co-expressed genes in LUAD (A), LUSC (B), MESO (C), OSCC (D), OV (E), PAAD (F), PCPG (G), PRAD (H), and READ (I), are presented as co-expression heatmaps (High: >median Low: <median). **p*<0.05, ***p*<0.01, ***p<0.001.

**Figure S13-4. Differential expression of** **DEPDC1** **co-expressed genes in 36 cancer types.** The expression levels of DEPDC1 co-expressed genes in SARC (A), SKCM (B), STAD (C), TGCT (D), THCA (E), THYM (F), UCEC (G), UCS (H), and UVM (I) are presented as co-expression heatmaps grouped (High: >median Low: <median). **p*<0.05, ***p*<0.01, ***p<0.001.

**Figure S14-1. Correlation of DEPDC1 co-expressed genes with 24 immune cell types by the ssGSEA algorithm in 36 cancers.** ACC (A), BLCA (B), BRCA (C), CESC (D), CHOL (E), DLBC (F), ESAD (G), ESCA(H) and ESCC (I),*P < 0.05, **P < 0.01, ***P < 0.001.

**Figure S14-2. Correlation of DEPDC1 co-expressed genes with 24 immune cell types by the ssGSEA algorithm in 36 cancers.** GBM (A), HNSC (B), KICH (C), KIRC (D), KIRP (E), LAML (F), LGG (G), LIHC(H) and LUAD (I),*P < 0.05, **P < 0.01, ***P < 0.001.

**Figure S14-3. Correlation of DEPDC1 co-expressed genes with 24 immune cell types by the ssGSEA algorithm in 36 cancers.** LUSC (A), MESO (B), OSCC (C), OV (D), PAAD (E), PCPG (F), PRAD (G), READ (H) and SARC (I),*P < 0.05, **P < 0.01, ***P < 0.001.

**Figure S14-4. Correlation of DEPDC1 co-expressed genes with 24 immune cell types by the ssGSEA algorithm in 36 cancers.** SKCM (A), STAD (B), TGCT (C), THCA (D), THYM (E), UCEC (F), COAD (G), UCS (H) and UVM (I),*P < 0.05, **P < 0.01, ***P < 0.001.

**Figure S15.** **Genetic alteration landscape of** **DEPDC1-related genes.** (A) Deleterious mutation frequency of related genes in each cancer type. (B) The mutation distribution of DEPDC1-related genes in different caner types. (C) The correlation of methylation with mRNA expression of DEPDC1-related genes in pan-cancers.

**Figure S16.** **CNV and methylation information about DEPDC1-related genes.** The Copy number variation (A), methylation differences expression (B) of DEPDC1-related genes in pan-cancers.

**Figure S17. Enrichment and Survival analysis of DEPDC1 co-expressed genes.** (A) GO and KEGG enrichment analysis of the co-expression genes of DEPDC1 via the Metascape database. (B) Network of enriched terms colored by cluster identity, where nodes that share the same cluster identity are typically close to each other. (C) The gene-gene interaction network for co-expression genes of DEPDC1 was analyzed using the GeneMANIA database. Each node represents a gene. The node color represents the possible functions of the respective gene. (D) OS, (E)PFI, (F) DSS, (G)DFI Univariate cox regression analysis of DEPDC1 co-expressed genes in pan-carcinoma. p-value > 0.05 is gray; p-value < 0.05, HR > 1 is red, HR < 1 is blue.

**Figure S18. DNA methylation of DEPDC1 in cancers.** (A) DNA methylation analysis in 33 cancer types. A red ID probe indicates that the probe targeted to the promotor region. (B) The correlation of DEPDC1 expression and methylation level of the CpG sites cg19634693 are shown in distinct cancers. The analysis here is based on TCGA and Infinium HumanMethylation450 BeadChip arrays.

**Figure S19. Potential transcription factors regulating DEPDC1 expression.** The upstream transcription factors (TFs) of DEPDC1 are predicted by eight public databases, including HumanTFDB, Gene card, JASPAR, ChIP Atlas, CistromeDB Toolkit, ENCODE, GTRD and hTFtarget. The TFs predicted by ≥ 3 databases were selected for further analysis. The experimentally validated TFs, which can up-/down-regulate DEPDC1 expression, were identified by means of the KnockTF2.0 database. We then generated lists of TFs, which overlap between predicted and experimentally validated TFs.

**Figure S20. Potential miRNAs targeting DEPDC1.** miRNAs that can silence DEPDC1 expression were predicted by six databases, including miRWalk, DIANA-TarBase v9.0, DIANA-MicroT-CDS, miRDB, StarBase and TargetScanHuman_8.0. The miRNAs predicted by ≥ 3 databases were selected for further analysis. Experimentally validated miRNAs were identified by means of the DIANA-Tarbase v8.0 and MiRtarbase databases. Overlaps between predicted and validated miRNAs are represented in a Venn diagram.

**Figure S21. DEPDC1 positively correlates with the enrichment of TH2 cells in different cancers.** The analysis of TCGA datasets identified significantly positive correlation of DEPDC1 expression and Th2 cell enrichment in 36 cancer types.

**Figure S22. Significant enrichment of TH2 cells in cancers that are DEPDC1 high rather than PRC1 low.** Transcripts from Th2 cells are particularly abundant in DEPDC1high expression compared with low expression tumor groups in 36 cancer types. The differential enrichment results of Th2 cells based on DEPDC1 expression are separately shown in the following cancers: ACC, BRCA, BLCA, CESC, COAD, CHOL, DLBC, ESAD, ESCA, ESCC, GBM, HNSC, KICH, KIRC, KIRP, LAML, LGG, LUAD, LUSC, LIHC, MESO, OV, OSCC, PAAD, PCPG, PRAD, READ, SARC, SKCM, STAD, TGCT, THCA, THYM, UCEC, UCS and UVM.**p*<0.05, ***p*<0.01 and ****p*<0.001.

**Figure S23. Survival analysis based on the combination of DEPDC1 expression and TH2 cell enrichment.** TCGA-based evaluation on prognostic value of DEPDC1 expression combined with Th2 cell enrichment in certain cancers. The survival curves were obtained using the TIMER2.0 online tool.

**Figure S24-1.**  **Relationship between** **Immunomodulatory factor and DEPDC1.** (A) immune checkpoint, (B) chemokines and (C) chemokine receptors genes *p<0.05, **p<0.01 and ***p<0.001.

**Figure S24-2.**  **Relationship between** **Immunomodulatory factor and DEPDC1.** (A) immune checkpoint, (B) chemokines and (C) chemokine receptors genes *p<0.05, **p<0.01 and ***p<0.001.

A

**Figure S25. Drug sensitivity analysis of DEPDC1.** (A) Spearman correlation between DEPDC1 and chemotherapy drugs in pan-cancer. (B) In pan-cancer, potential small molecules and drugs to correct biological effects caused by dysregulation of DEPDC1 expression using the XSum algorithm (from the Cmap database).
